# miR-195 competes with HuR to modulate *stim1* mRNA stability and regulate cell migration

**DOI:** 10.1093/nar/gkt565

**Published:** 2013-06-25

**Authors:** Ran Zhuang, Jaladanki N. Rao, Tongtong Zou, Lan Liu, Lan Xiao, Shan Cao, Natasha Z. Hansraj, Myriam Gorospe, Jian-Ying Wang

**Affiliations:** ^1^Department of Surgery, Cell Biology Group, University of Maryland School of Medicine, MD 21201, USA, ^2^Baltimore Veterans Affairs Medical Center, Baltimore, MD 21201, USA, ^3^Department of Pathology, University of Maryland School of Medicine, MD 21201, USA and ^4^Laboratory of Genetics, National Institute on Aging-IRP, NIH, Baltimore, MD 21224, USA

## Abstract

Stromal interaction molecule 1 (Stim1) functions as a sensor of Ca^2+^ within stores and plays an essential role in the activation of store-operated Ca^2+^ entry (SOCE). Although lowering Stim1 levels reduces store-operated Ca^2+^ entry and inhibits intestinal epithelial repair after wounding, the mechanisms that control Stim1 expression remain unknown. Here, we show that cellular Stim1 abundance is controlled posttranscriptionally via factors that associate with 3′-untranslated region (3′-UTR) of *stim1* mRNA. MicroRNA-195 (miR-195) and the RNA-binding protein HuR competed for association with the *stim1* 3′-UTR and regulated *stim1* mRNA decay in opposite directions. Interaction of miR-195 with the *stim1* 3′-UTR destabilized *stim1* mRNA, whereas the stability of *stim1* mRNA increased with HuR association. Interestingly, ectopic miR-195 overexpression enhanced *stim1* mRNA association with argonaute-containing complexes and increased the colocalization of tagged *stim1* RNA with processing bodies (P-bodies); the translocation of *stim1* mRNA was abolished by HuR overexpression. Moreover, decreased levels of Stim1 by miR-195 overexpression inhibited cell migration over the denuded area after wounding but was rescued by increasing HuR levels. In sum, Stim1 expression is controlled by two factors competing for influence on *stim1* mRNA stability: the mRNA-stabilizing protein HuR and the decay-promoting miR-195.

## INTRODUCTION

Store-operated Ca^2+^ entry (SOCE) is an important process in cellular physiology that serves essential functions ranging from regulation of gene transcription to cell motility (1,2). In non-excitable cells, such as intestinal epithelial cells (IECs), canonical transient receptor potential-1 (TRPC1) is thought to function as a Ca^2+^-permeable channel mediating SOCE and regulates IEC migration after injury (3–5). Although the exact mechanisms control the activity of TRPC1 and other SOC channels are still unclear, the single membrane-spanning protein stromal interaction molecule 1 (Stim1) has been identified as the Ca^2+^ sensor in the endoplasmic reticulum and plays a crucial role in the activation of TRPC1- and Orai1-mediated Ca^2+^ influx after store depletion (6–8). Stim1 is predominantly located in the endoplasmic reticulum in unstimulated cells, but it rapidly translocates to the plasma membrane after Ca^2+^ store depletion or in response to various biological stimuli (9–11). In the plasma membrane, Stim1 interacts with and activates TRPC1 and Orai1 channels, thereby increasing SOCE. Inhibition of the expression of Stim1 reduces SOC activation, whereas overexpression of a constitutively active EF-hand motif mutant Stim1 increases its levels at the plasma membrane region and enhances SOCE (12,13). Our previous studies show that Stim1 translocation to the plasma membrane in IECs increases after wounding, and that the induced membrane Stim1 stimulates intestinal epithelial repair by enhancing TRPC1-mediated Ca^2+^ signaling (14,15). However, the molecular mechanism underlying control of cellular Stim1 abundance, especially at the posttranscription level, is unknown.

The regulation of mRNA stability and translation is a major mechanism by which mammalian cells control gene expression in response to changes in environmental conditions (16,17). mRNAs are targeted for rapid degradation and/or translational repression through a process involving the interaction of specific mRNA sequences (*cis*-elements) with specific *trans*-acting factors such as microRNAs (miRNAs) and RNA-binding proteins (RBPs) (18,19). miRNAs are a class of small non-coding RNAs spanning ∼22 nt, which regulate a variety of cellular processes (20,21). The study of miRNA biology has attracted remarkable attention during the past decade (22–25). Generally, miRNAs function by binding to the 3′-untranslational regions (UTRs) of target mRNAs, destabilizing them and/or inhibiting their translation (21,24,25). Although the exact functions of miRNAs in human development and physiology are not fully elucidated, their differential expression of in certain diseases has been linked to several human pathologies. Recently, miRNAs have also emerged as master regulators of the maintenance of the homeostasis of normal gastrointestinal mucosa (26,27), the tissue with the most rapid turnover rate in the body (28). The evolutionarily conserved microRNA-195 (miR-195) is highly abundant in normal gastrointestinal mucosa, although its levels decrease significantly in cancer tissues (29–31). miR-195 inhibits cell proliferation by targeting the cyclin-dependent kinase 4 (CDK4), cyclin D1, CDK6 and WEE1 (30–32), promotes apoptosis by downregulating Sirt1 (33) and affects cell invasion by modulating ActRIIA expression (34).

Although many RBPs have housekeeping functions and interact with many cellular transcripts, several RBPs associate with specific subsets of mRNAs and play important roles in controlling gene expression patterns in response to stress (35–38). HuR is among the most prominent sequence-specific translation and turnover regulatory RBPs. HuR has two N-terminal RNA-recognition motifs through which it associates with high affinity and specificity with AU-rich elements located in the 3′-UTRs of labile mRNAs (39,40). Through these interactions, HuR increases the stability and modulates the translation of target transcripts (35–38). Several studies have also revealed that RBPs and miRNAs can function jointly to regulate shared target mRNAs (27,41,42). For example, HuR recruits let-7/RNA-induced silencing complex to repress the translation of *MYC* mRNA (43), whereas the RBP Dnd-1 inhibits miRNA access to target mRNA (44). We have recently demonstrated that CUG-binding protein 1 (CUGBP1) and miR-222 synergistically repress CDK4 mRNA translation (27).

An *en masse* search for miR-195 and HuR target mRNAs identified the *stim1* mRNA as a putative target of both miR-195 and HuR, as there are several computationally predicted miR-195 binding sites and HuR-hit motifs in the *stim1* 3′-UTR. Here, we sought to investigate the molecular mechanism that governs Stim1 expression by miR-195 and HuR and to further study their influence on epithelial repair after wounding. Our results show that miR-195 interacts with and destabilizes *stim1* mRNA, whereas HuR competes with miR-195 for association with the *stim1* 3′-UTR, thus stabilizing *stim1* mRNA. Moreover, miR-195 increases the association of *stim1* mRNA with processing bodies (P-bodies), where mRNAs are sorted for degradation. In contrast, HuR blocks *stim1* mRNA translocation to P-bodies by displacing miR-195. The miR-195/HuR-modulated Stim1 expression plays an important role in the regulation of epithelial repair, as increased miR-195 repressed cell migration after wounding while HuR restored it.

## MATERIALS AND METHODS

### Chemicals and cell culture

Tissue culture medium and dialyzed fetal bovine serum were from Invitrogen (Carlsbad, CA), and biochemicals were from Sigma (St. Louis, MO). The antibodies recognizing Stim1, HuR and GAPDH were obtained from Santa Cruz Biotechnology (Santa Cruz, CA) and BD Biosciences, and the secondary antibody conjugated to horseradish peroxidase was from Sigma. Pre-miR^TM^ miRNA precursor and anti-miR^TM^ miRNA inhibitor of miR-195 were purchased from Ambion (Austin, TX). Biotin-labeled miRNA-195 was custom made by Dharmacon (Lafayette, CO). The IEC-6 cell line, derived from normal rat intestinal crypt cells (45), was used at passages 15–20; cells were maintained in Dulbecco’s modified Eagle’s medium supplemented with 5% heat-inactivated fetal bovine serum.

### Plasmid construction

Recombinant adenoviral plasmids containing human HuR were constructed by using the Adeno-X Expression System according to the protocol provided by the manufacturer (Clontech). Briefly, the full-length cDNA of human wild-type HuR was cloned into the pShuttle by digesting the BamHI/HindIII and ligating the resultant fragments into the XbaI site of the pShuttle vector (37). pAdeno-HuR (AdHuR) was constructed by digesting the pShuttle construct with PI-SceI/I-CeuI and ligating the resultant fragment into the PI-SceI/I-CeuI sites of the pAdeno-X adenoviral vector. Recombinant adenoviral plasmids were packaged into infectious adenoviral particles by transfecting human embryonic kidney 293 cells using LipofectAMINE Plus reagent (Gibco-Bethesda Res Lab, Gaithersburg, MD). Titers of the adenoviral stock were determined by standard plaque-forming assay. Recombinant adenoviruses were screened for the expression of the introduced gene by western blot analysis using anti-HuR antibody. pAdeno-X, which was the recombinant replication-incompetent adenovirus carrying no HuR cDNA insert (Adnull), was grown and purified as described earlier in the text and served as a control adenovirus. Cells were infected with AdHuR or Adnull, and expression of HuR was assayed at 24 or 48 h after the infection.

The chimeric firefly luciferase reporter construct containing the entire *stim1* cDNA was described previously (35,36). The full-length Stim1 5′-UTR, CR or 3′-UTR and different 3′-UTR fragments with or without predicted miR-195 binding sites were amplified and subcloned into the pmirGLO Dual-Luciferase miRNA Target Expression Vector (Promega, Madison, WI) to generate the pmirGLO-Luc-Stim1-5′UTR, pmirGLO-Luc-Stim1-CR and pmirGLO-Stim1-3′UTR. The sequence and orientation of the fragment in the luciferase reporter were confirmed by DNA sequencing and enzyme digestion. Transient transfections were performed using the Lipofectamine Reagent as recommended by the manufacturer (Invitrogen) (37,38). Luciferase activity was measured using the Dual Luciferase Assay System, and the levels of firefly luciferase activity were normalized to Renilla luciferase activity and were further compared with the levels of luciferase mRNA in every experiment. Both pcDNA-MS2 and pcDNA-MS2-YFP plasmids were described previously (46), and the fragment of *stim1* 3′-UTR was inserted into pcDNA-MS2 at the XhoI site. All of primer sequences for generating these constructs are provided in Supplementary Table S1.

### RT-PCR and real-time quantitative PCR analysis

Total RNA was isolated by using RNeasy mini kit (Qiagen, Valencia, CA) and used in reverse transcription and PCR amplification reactions as described (35). The levels of glyceraldehyde-3-phosphate dehydrogenase (*Gapdh*) PCR product were assessed to monitor the evenness in RNA input in RT-PCR samples. Real-time quantitative PCR (Q-PCR) analysis was performed using 7500-Fast Real-Time PCR Systems with specific primers, probes and software (Applied Biosystems, Foster City, CA). For miRNA studies, the levels of miRNA-195 were also quantified by Q-PCR by using Taqman MicroRNA assay; small nuclear RNA U6 was used as endogenous control.

### Western blot analysis

Whole-cell lysates were prepared using 2% SDS, sonicated and centrifuged (12 000 rpm) at 4°C for 15 min. The supernatants were boiled for 5 min and size fractionated by SDS–PAGE (7.5% acrylamide). After transferring proteins onto nitrocellulose filters, the blots were incubated with primary antibodies recognizing Stim1 or HuR; following incubations with secondary antibodies, immunocomplexes were developed by using chemiluminescence.

### Biotin labeled miR-195 pulldown assays

Biotin-labeled miR-195 was transfected, and 24 h later, whole-cell lysates were collected (27), mixed with Streptavidin-Dynal beads (Invitrogen, Carlsbad, CA) and incubated at 4°C with rotation overnight. After the beads were washed thoroughly, the bead-bound RNA was isolated and subjected for RT followed by Q-PCR analysis. Input RNA was extracted and served as control.

### Biotin pulldown assays and ribonucleoprotein immunoprecipitation analysis

The synthesis of biotinylated transcripts and analysis of RBPs bound to biotinylated RNA were carried out as previously described (47). Complementary DNA from IEC-6 cells was used as a template for PCR amplification of 5′-UTR, CR and 3′-UTR of *stim1* mRNA. The 5′ primers contained the T7 RNA polymerase promoter sequence [(T7), CCAAGCTTCTAATACGAC-TCACTATAGGGAGA]. All sequences of oligonucleotides for preparation of full-length *stim1* 5′-UTR, CR, 3′-UTR and various short RNA probes for mapping the *stim1* 3′-UTR were described in Supplementary Table S2. PCR-amplified products were used as templates to transcribe biotinylated RNAs by using T7 RNA polymerase in the presence of biotin-cytidine 5′-triphosphate as described (48). Biotinylated transcripts (6 µg) were incubated with 120 µg of cytoplasmic lysates for 30 min at room temperature. Complexes were isolated with paramagnetic streptavidin-conjugated Dynabeads (Dynal, Oslo, Norway) and analyzed by western blot analysis.

To assess the association of endogenous HuR with endogenous *stim1* mRNA, immunoprecipitation (IP) of ribonucleoprotein (RNP) complexes was performed as described (38,47). Twenty million cells were collected per sample, and lysates were used for IP for 4 h at room temperature in the presence of excess (30 µg) IP antibody (IgG, anti-HuR). RNA in IP materials was used in RT followed by PCR and Q-PCR analysis to detect the presence of *stim1* and *gapdh* mRNAs.

### Immunofluorescence staining

Immunofluorescence was performed as described (49). Cells were fixed using 3.7% formaldehyde and the rehydrated samples incubated overnight at 4°C with primary antibody anti-Ago2 or RCK (also known as DDX6-DEAD-box RNA helicase) diluted 1:300 in blocking buffer, then incubated with secondary antibody conjugated with Alexa Fluor-594 (Molecular Probes, Eugene, OR) for 2 h at room temperature. Images were processed using Axio Observer microscope (ZEISS) with LSM 510 Meta (ZEISS) image processing software.

### Measurement of cell migration

Migration assays were carried out as described earlier (5,50). Cells were plated at 6.25 × 10^4^/cm^2^ in Dulbecco’s modified Eagle’s medium containing FBS on 60-mm dishes thinly coated with Matrigel following the manufacturer’s instructions (BD Biosciences) and were incubated as described for stock cultures. Cells were fed on day 2, and cell migration was assayed on day 4. To initiate migration, the cell layer was scratched with a single-edge razor blade cut to ∼27 mm in length. The scratch was made over the diameter of the dish and extended over an area 7–10 mm wide. The migrating cells in six contiguous 0.1-mm squares were counted at ×100 magnification beginning at the scratch line and extending as far out as the cells had migrated. All experiments were carried out in triplicate, and the results were reported as number of migrating cells per millimeter of scratch.

### Statistics

Values are the means ± SE from three to six samples. Western blot analysis and immunofluorescence staining were repeated three times. The significance of the difference between means was determined by ANOVA. The level of significance was determined by using Duncan’s multiple-range test (51).

## RESULTS

### miR-195 directly interacts with and destabilizes *stim1* mRNA

**Figure 1.**
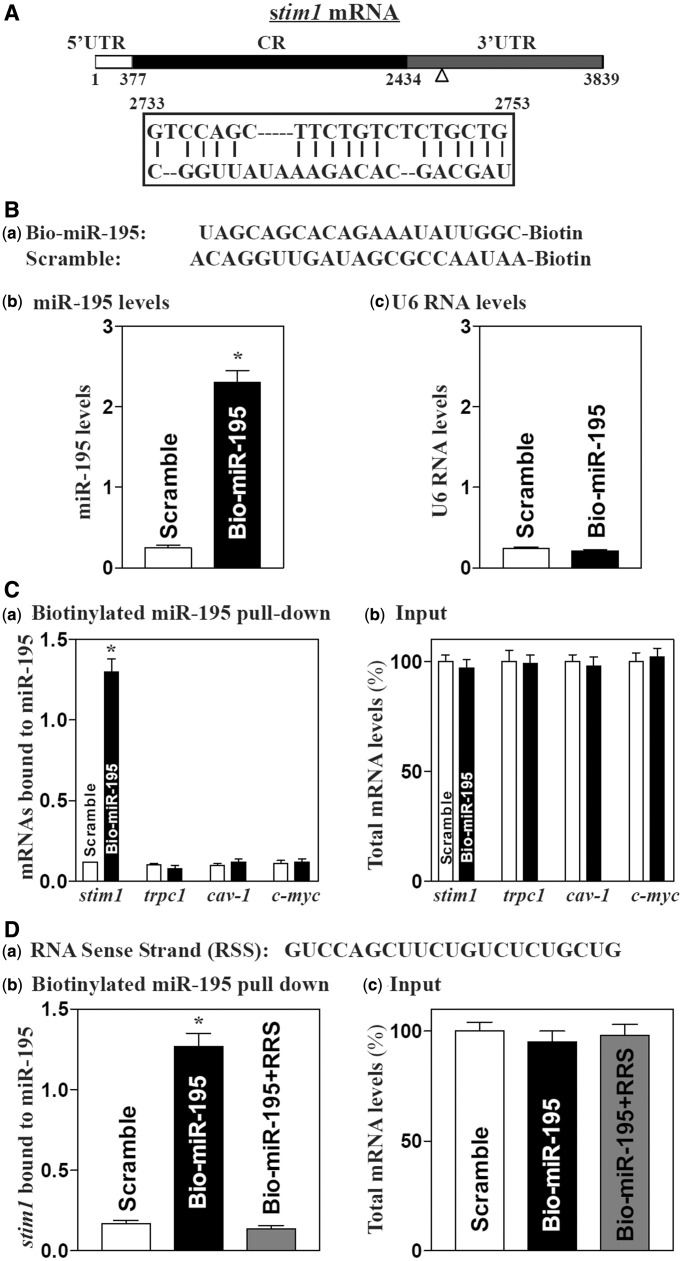
miR-195 associates with the *stim1* mRNA. (**A**) Schematic representation of the *stim1* mRNA depicting target site for miR-195 in its 3′-UTR. Alignment of the *stim1* mRNA sequence with miR-195: top strand, *stim1* mRNA; bottom strand, miR-195. (**B**) Levels of biotinylated miR-195 after transfection for 24 h: (**a**) schematic representation of biotinylated miR-195 and scramble oligomer; (**b**) miR-195 levels as measured by Q-PCR analysis; and (**c**) U6 RNA levels. Values are the means ± SEM from three separate experiments. *P < 0.05 compared with cells transfected with control scramble oligomer. (**C**) Binding of biotinylated miR-195 to mRNAs encoding Stim1, TRPC1, Cav-1 and c-Myc: (**a**) levels of mRNA in the materials pulled down by biotin-miR-195; and (**b**) levels of total input mRNAs. *P < 0.05 compared with cells transfected with control scramble oligomer. (**D**) Changes in the levels of miR-195–*stim1* mRNA association after addition of fraction of *stim1* mRNA sense strand to the reaction medium: (**a**) schematic representation of *stim1* mRNA sense strand (RSS); (**b**) levels of mRNA in the materials pulled down by biotin-miR-195; and (**c**) levels of total input mRNAs. *P < 0.01 compared with cells transfected with scrambled oligomer.

The *stim1* mRNA is a potential target of miR-195, as it contains one computationally predicted miR-195 site in its 3′-UTR (Figure 1A), using standard online software (TargetScan and RNA22). To test the involvement of miR-195 in the regulation of Stim1 expression, we first examined the association of miR-195 with the *stim1* mRNA by RNA pull-down assays using biotin-labeled miR-195 (Dharmacon, shown in Figure 1Ba). Twenty-four hours after the transfection, miR-195 levels increased significantly, but the levels of the housekeeping non-coding RNA U6 did not (Figure 1Bb and c). The *stim1* mRNA was enriched in the materials pulled down by biotin-miR-195, but not from cells transfected with control scramble RNA (Figure 1Ca). The association of miR-195 with the *stim1* mRNA was specific, as increasing the levels of biotin-miR-195 did not increase its interaction with the mRNAs encoding TRPC1, caveolin 1 (CAV1) and c-Myc. In addition, transfection with biotin-labeled miR-195 failed to alter the steady–state levels of total *stim1*, *trpc1*, *cav1* and *myc* mRNAs (Figure 1Cb). We also examined the interaction of miR-222 with the *stim1* mRNA and found that increasing the levels of biotin-miR-222 did not alter its association with the *stim1* mRNA (Supplementary Figure S1). Furthermore, association of miR-195 with the *stim1* mRNA was completely inhibited when unlabeled synthetic RNA sense sequence (RSS) from Stim1 3′-UTR (positions from 2733 to 2753 with the miR-195-binding site, Figure 1Da) was added to cell culture medium together with biotin-labeled miR-195 (Figure 1Db). Treatment with RSS had no effect on the levels of total *stim1* mRNA (Figure 1Dc). These results strongly suggest that miR-195 directly interacts with the *stim1* mRNA via the *stim1* 3′-UTR.

**Figure 2.**
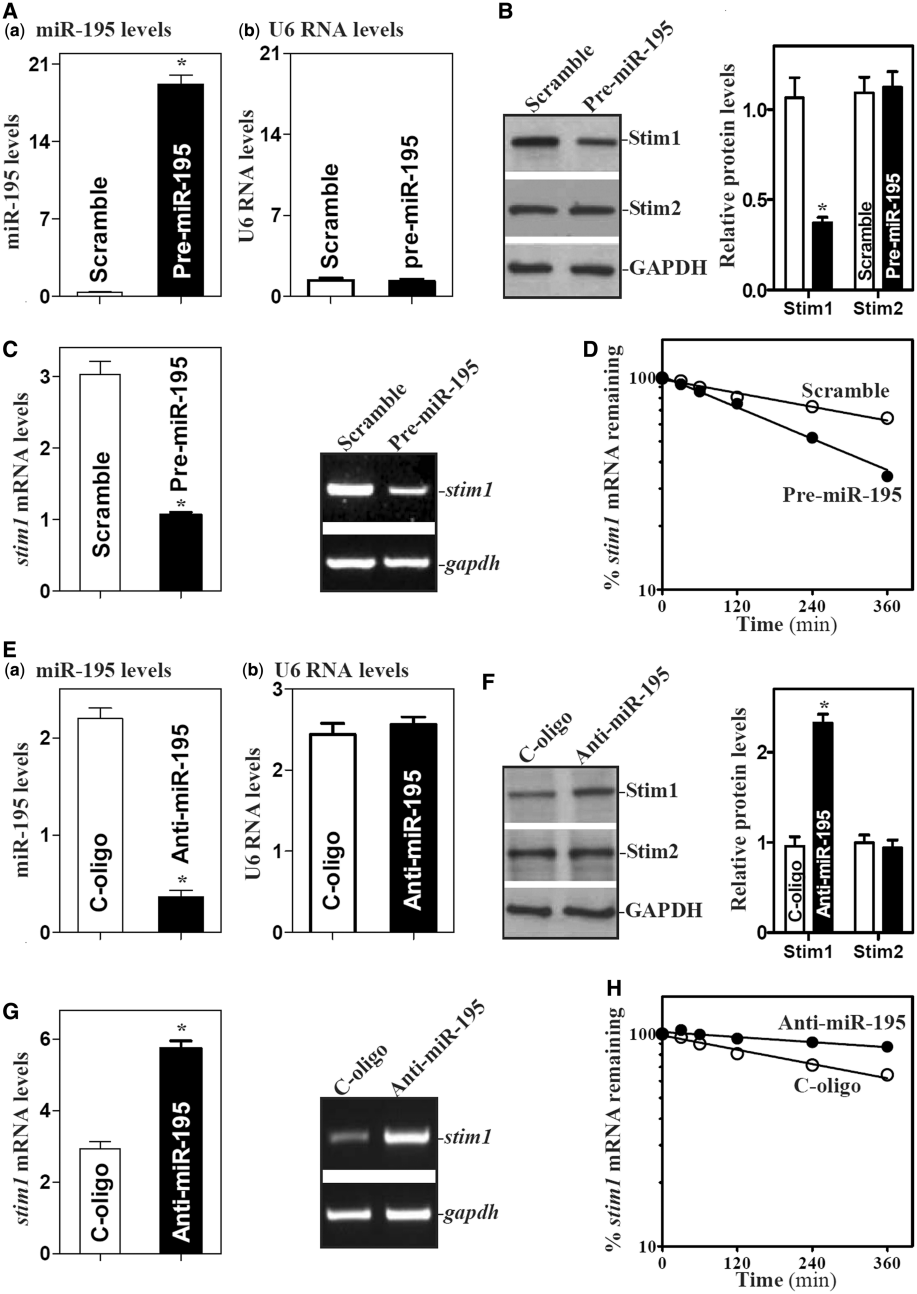
miR-195 represses Stim1 expression by destabilizing *stim1* mRNA. (**A**) Levels of miR-195 in cells transfected with pre-miR-195 for 48 h as measured by Q-PCR analysis: (**a**) miR-195 levels; and (**b**) U6 RNA levels. Values are the means ± SEM from three separate experiments. *P < 0.05 compared with cells transfected with control scramble oligomer. (**B**) Changes in the levels of Stim1 and STIM2 proteins after ectopic miR-195 overexpression. Whole-cell lysates were prepared for western blotting; equal loading was monitored by assessing GAPDH levels. (**C**) Levels of *stim1* mRNA as examined by Q-PCR (*left*) or RT-PCR (*right*) analyses. (**D**) Half-life of *stim1* mRNA in cells overexpressing miR-195 as measured by Q-PCR analysis. Total cellular RNA was isolated at indicated times after administration of actinomycin D (5 µg/ml), and the remaining levels of *stim1* and *Gapdh* mRNAs were measured by Q-PCR analysis. (**E–H**) Effect of miR-195 silencing on Stim1 expression. Forty-eight hours after cells were transfected with the corresponding oligomer targeting miR-195 (anti-miR-195) or control oligomer (C-oligo), various measurements were performed as described earlier in the text. Values are the means ± SEM from three separate experiments. *P < 0.01 compared with cells transfected with C-oligo.

Second, we investigated the functional consequence of (miR-195-*stim1* mRNA) association. The levels of miR-195 were increased by transfection with the miR-195 precursor (pre-miR-195) or decreased by transfecting the corresponding antisense oligomer (antagomir) targeting miR-195 (anti-miR-195). By 48 h after transfection, pre-miR-195 levels increased strongly and specifically (Figure 2Aa,b), while it decreased Stim1 protein levels (Figure 2B; without affecting STIM2 protein levels). Increasing miR-195 levels also decreased *stim1* mRNA abundance (Figure 2C) as a result of increased degradation rate, since the *stim1* mRNA half-life was reduced in cells overexpressing miR-195 compared with that measured in control cells (Figure 2D). In contrast, decreased levels of endogenous miR-195 by transfecting IEC-6 cells with an antagomir targeting miR-195 (anti-miR-195, Figure 2E) increased Stim1 expression, as indicated by the increased levels of Stim1 protein (Figure 2F) and *stim1* mRNA (Figure 2G) in anti-miR-195-transfected cells. Anti-miR-195 increased the stability of *stim1* mRNA, since miR-195 silencing increased half-life of the *stim1* mRNA (Figure 2H). Together, these results indicate that miR-195 represses Stim1 expression by destabilizing the *stim1* mRNA.

**Figure 3.**
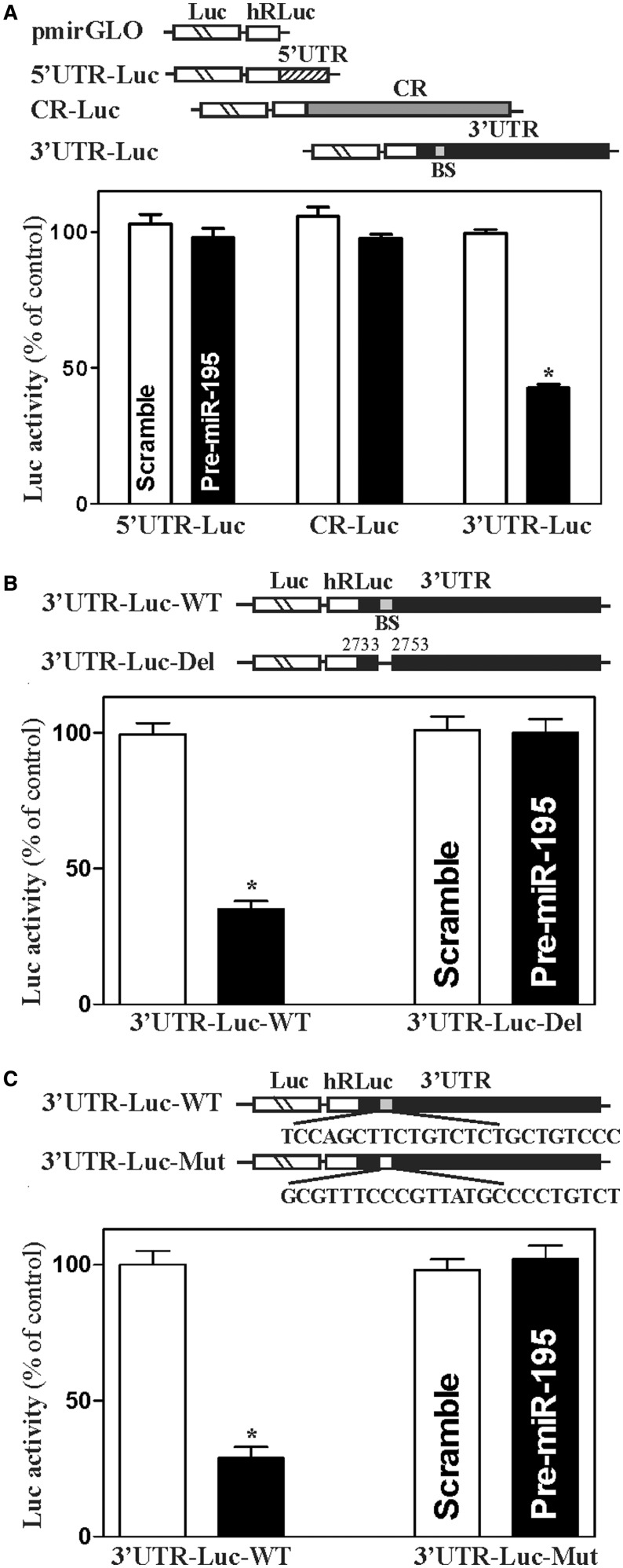
Deletion of miR-195 binding site in *stim1* 3′UTR prevents miR-195-mediated repression of Stim1. (**A**) Levels of reporter activities as measured by analysis of *stim1* 5′UTR, CR or 3′luciferase reporters after ectopic overexpression of miR-195. Top, schematic of plasmids of different chimeric firefly luciferase *stim1* reporters. BS, predicted miR-195-binding site. Bottom, levels of activities of luciferase reporters containing *stim1* 5′UTR, CR or 3′UTR. Twenty-four hours after transfection with pre-miR-195, cells were transfected with different *stim1* luciferase reporter plasmids. Levels of firefly and *Renilla* luciferase activities were assayed 16 h later. Results were normalized to the *Renilla* luciferase activities and expressed as the means ± SEM data from three separate experiments. *P < 0.05 compared with cells transfected with control scrambled oligomer. (**B**) Effect of deletion of specific miR-195 binding site (schematic) in *stim1* 3′UTR on luciferase reporter activities after ectopic miR-195 overexpression. *P < 0.05 compared with cells transfected with control scrambled oligomer. (**C**) Effect of point-mutation of specific miR-195 binding site (schematic) in *stim1* 3′UTR on luciferase reporter activities after ectopic miR-195 overexpression. *P < 0.05 compared with cells transfected with control scrambled oligomer.

Third, we determined whether destabilization of the *stim1* mRNA by miR-195 was mediated through the *Stim1* 5′-UTR, CR, or 3′-UTR. Fractions of the *stim1* 5′-UTR, CR and 3′-UTR were sub-cloned into the pmirGLO dual-luciferase miRNA target expression vector to generate pmirGLO-Stim1-5′UTR, pmirGLO-Stim1-CR and pmirGLO-Stim1-3′UTR reporter constructs (Figure 3A, schematic). miR-195 overexpression by transfection with pre-miR-195 selectively decreased the levels of pmirGLO-Stim1-3′UTR luciferase reporter activity (Figure 3A, *bottom*), but it failed to inhibit the activities of pmirGLO-Stim1-5′-UTR and -CR reporters. Consistently, the half-life of luciferase mRNA expressed from the pmirGLO-Stim1-3′UTR luciferase reporter construct was also decreased by transfection with pre-miR-195 (Supplementary Figure S2). These findings strongly suggest that increasing the levels of miR-195 destabilizes *stim1* mRNA through the *stim1* 3′-UTR. To further characterize the specific binding site of miR-195 in the Stim1 3′-UTR, various reporter constructs that expressed chimeric RNA containing the luciferase and partial transcripts spanning the *stim1* 3′UTR with or without the potential binding site were prepared (Supplementary Figure S3). Reporters bearing a deletion or mutation of the *stim1* 3′-UTR were also prepared by eliminating nucleotides spanning positions 2733-2753 of the *stim1* 3′-UTR (Figure 3B, schematic) or by mutating the site (Figure 3C, schematic). Ectopic miR-195 overexpression decreased the levels of luciferase reporter gene activity when cells were transfected with the 3′UTR-Luc (full-length 3′-UTR) and 3′UTR-F1-L (containing the miR-195 binding site), but repression by miR-195 was completely prevented when this specific site from the *stim1* 3′-UTR was deleted or mutated (Figure 3B,C, *bottom*; Supplementary Figure S3). To verify the interaction of miR-195 with the reporter mRNA containing the *stim1* 3′-UTR, the binding of miR-195 to the reporter mRNA expressed from pmirGLO-Stim1-5′UTR, pmirGLO-Stim1-CR and pmirGLO-Stim1-3′UTR constructs was examined by using biotin-labeled miR-195 pulldown assays. As expected, miR-195 only associated with the reporter mRNA containing *stim1* 3′-UTR but not with those containing *stim1* 5′-UTR or CR; this interaction was abolished by deletion mutation and point mutation of the miR-195 binding site from the 3′-UTR (Supplementary Figure S4). Taken together, these results indicate that miR-195 interacts with *stim1* mRNA via the specific binding site at 2733-2753, thus destabilizing *stim1* mRNA.

### HuR stabilizes *stim1* mRNA by interacting with *stim1* 3′-UTR

**Figure 4.**
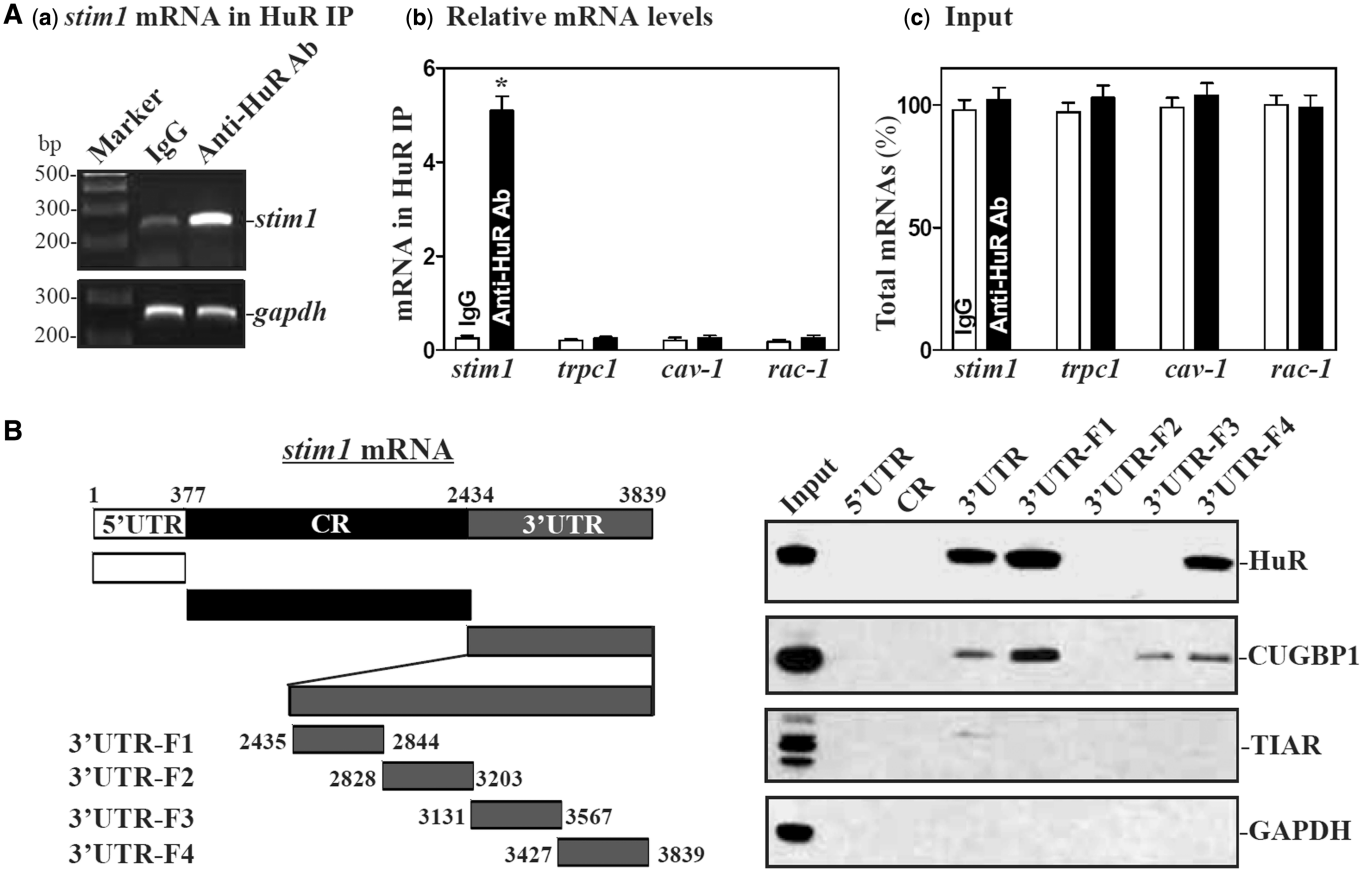
HuR binds to the *stim1* 3′UTR. (**A**) Association of endogenous HuR with endogenous Stim1 mRNA. After IP of RNA-protein complexes from cell lysates using either anti-HuR antibody (Ab) or control IgG, RNA was isolated and used in RT reactions. (**a**) RT-PCR product of Stim1 was visualized in ethidium bromide-stained agarose gels; (**b**) levels of mRNAs in HuR IP or IgG IP materials as measured by Q-PCR analysis; and (**c**) levels of total input mRNAs. Values are the means ± SEM from triplicate samples. *P < 0.05 compared with IgG IP. (**B**) Representative HuR, CUGBP1 and TIAR immunoblots using the pull-down materials by biotinylated transcripts of *stim1* 5′UTR, CR, 3′UTR and different fragments of 3′UTR. Left panel shows schematic representation of various *stim1* biotinylated transcripts used in this study. Cytoplasmic lysates were incubated with 6 µg of biotinylated *stim1* 5′UTR, CR, 3′UTR and fragments of *stim1* 3′UTR for 30 min at 25°C, and the resulting RNP complexes were pulled down by streptavidin-coated beads. The presence of HuR, CUGBP1 or TIAR in the pull-down material was assayed by western blotting. GAPDH in the pull-down material was also examined and served as a negative control.

Increasing evidence shows that miRNAs and RBPs such as HuR jointly regulate expression of shared target mRNAs either synergistically or antagonistically (27,41–43). Since there are several computationally predicted hits of HuR motif in the *stim1* mRNA, we further investigated the influence of HuR on miR-195-mediated repression of Stim1 expression. First, we examined if *stim1* mRNA associated with HuR by performing RNP IP assays using anti-HuR antibody under conditions that preserved RNP integrity (42). The interaction of *stim1* mRNA with HuR was examined by isolating RNA from the IP material and subjecting it to reverse transcription (RT) followed by either conventional PCR or Q-PCR analyses. As shown in Figure 4Aa,b, the *stim1* PCR products were highly enriched in HuR samples compared with control IgG samples, while HuR did not preferentially bind to trpc1, cav1, or rac1 mRNAs.

HuR-*stim1* mRNA associations were further tested by using biotinylated transcripts which spanned the *stim1* mRNA regions shown (Figure 4B, schematic). Following incubation with cytoplasmic lysates, the interaction between the biotinylated *stim1* transcripts and HuR was examined by biotin pulldown followed by Western blot analysis (47,48). As shown, HuR readily associated with the *stim1* 3′-UTR but not with its 5′-UTR and CR transcripts. The *stim1* 3′-UTR also interacted with other RBPs such as CUGBP1 but did not bind to T cell-restricted intracellular antigen 1-related protein (TIAR). Further mapping of the interaction between HuR and *stim1* 3′-UTR was performed by testing the interaction of partial biotinylated transcripts spanning the *stim1* 3′-UTR (Supplementary Figure S5A, schematic) with HuR using pull down assays. HuR was found to bind specifically to fragments 3′UTR-F1 and 3′UTR-F4, the partial transcripts which contained hits of the HuR signature motif (Supplementary Figure S5A and B). In contrast, there was no detectable binding of HuR to fragments 3′UTR-F2 and 3′UTR-F3, which lacked putative HuR hits. These findings indicate that HuR specifically associates with the s*tim1* 3′-UTR.

**Figure 5.**
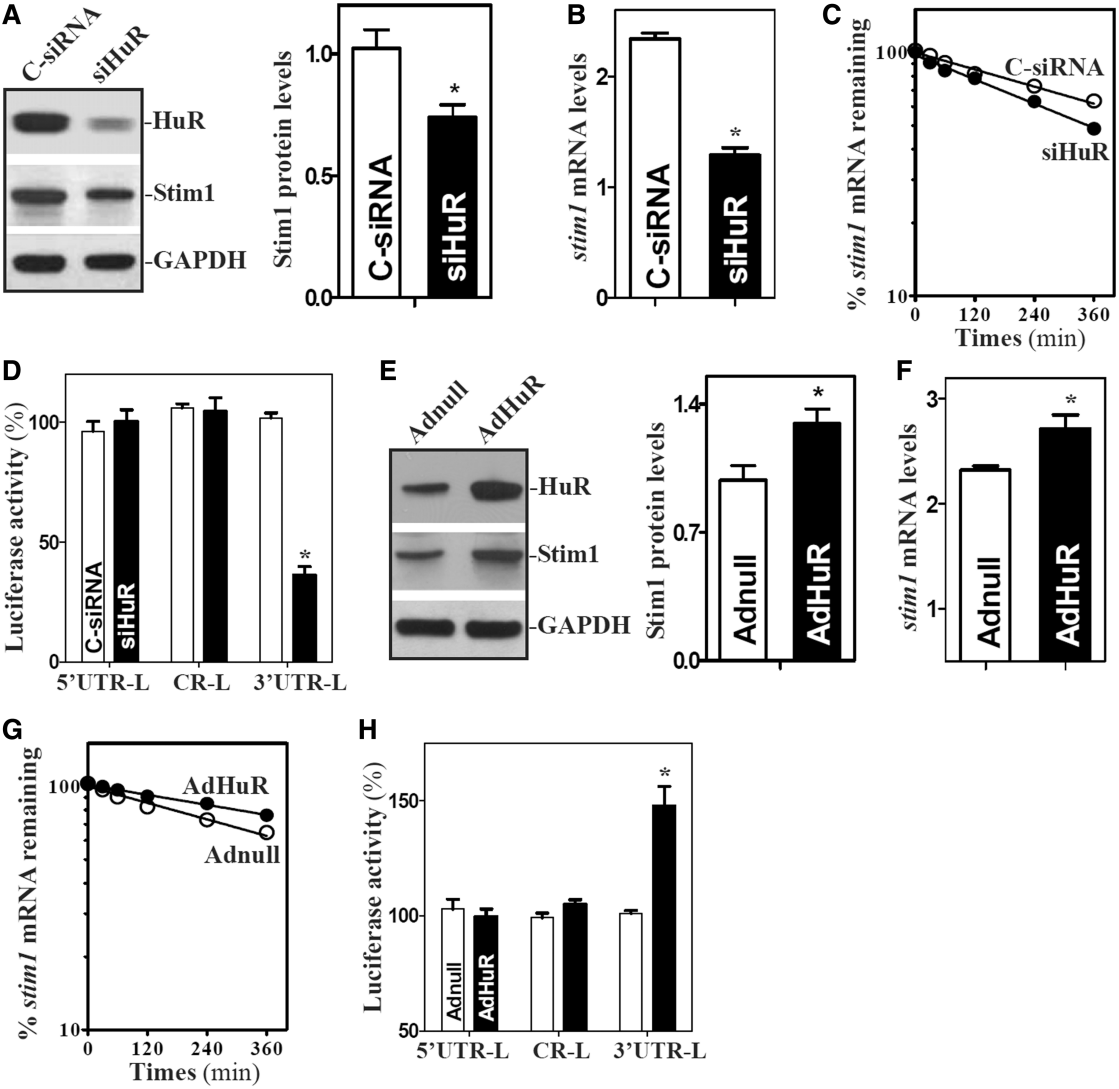
HuR enhances Stim1 expression by stabilizing *stim1* mRNA. (**A**) The effect of HuR silencing on Stim1 protein expression. Left panel, representative immunoblots of HuR and Stim1 proteins in HuR-silenced cells. After cells were transfected with either siRNA targeting the HuR mRNA-coding region (siHuR) or control siRNA (C-siRNA) for 48 h, whole cell lysates were harvested for western blotting analysis. Right panel, quantitative analysis of the immunoblotting signals as measured by densitometry. Values were the means ± SEM of data from triplicate experiments. *P < 0.05 compared with cells transfected with C-siRNA. (**B**) Levels of *stim1* mRNA in cells treated as described in (**A**). Total RNA was harvested, and the levels of *stim1* mRNA were measured by Q-PCR analysis. Data were normalized to *Gapdh* mRNA level, and values are the means ± SEM of data from triplicate experiments. (**C**) Half-life of *stim1* mRNA in cells described in (A). (**D**) Changes in activities of luciferase reporters containing *stim1* 5′UTR, CR or 3′in cells described in (A). *P < 0.05 compared with cells transfected with control siRNA. (**E–H**) Effect of ectopic overexpression of HuR on Stim1 expression. Cells were infected with the recombinant adenoviral vector encoding HuR cDNA (AdHuR) or adenoviral vector lacking HuR cDNA (Adnull) for 48 h; various measurements were performed as described earlier in the text. Values are the means ± SEM of data from triplicate experiments. *P < 0.05 compared with cells infected with Adnull.

Second, we determined whether HuR-*stim1* mRNA associations altered Stim1 expression. Consistent with our previous studies (37,38), transfection with small interfering RNA (siRNA) targeting the HuR mRNA (siHuR) decreased HuR protein levels by >80%; this reduction was specific, as other RBPs such as CUGBP1 and TIAR were not affected in HuR-silenced cells as reported previously (36). Importantly, HuR silencing reduced Stim1 protein by ∼30% (Figure 5A, right) and *stim1* mRNA levels (Figure 5B). These reductions were due to the destabilization of *stim1* mRNA, as silencing HuR selectively lowered the *stim1* mRNA half-life (Figure 5C). HuR regulates the stability of *stim1* mRNA by interacting with its 3′-UTR, as HuR silencing decreased the levels of Stim1-3′UTR luciferase reporter activity (Figure 5D), but not the activities of Stim1 5′-UTR-Luc and Stim1-CR-Luc reporter genes. On the other hand, ectopic overexpression of HuR by infection with the adenoviral vector containing the corresponding HuR cDNA (AdHuR) increased the Stim1 expression (Figure 5E and F) by stabilizing *stim1* mRNA (Figure 5G) via interaction with its 3′-UTR (Figure 5H). The levels of Stim1 protein and *stim1* mRNA in cells overexpressing HuR was ∼1.2-fold of those observed in cells infected with Adnull. In contrast, neither HuR protein levels nor Stim1 expression were altered by infection with the control adenovirus (Adnull). These results indicate that HuR enhances Stim1 expression by stabilizing the *stim1* mRNA.

### miR-195 and HuR regulate *stim1* mRNA stability antagonistically

**Figure 6.**
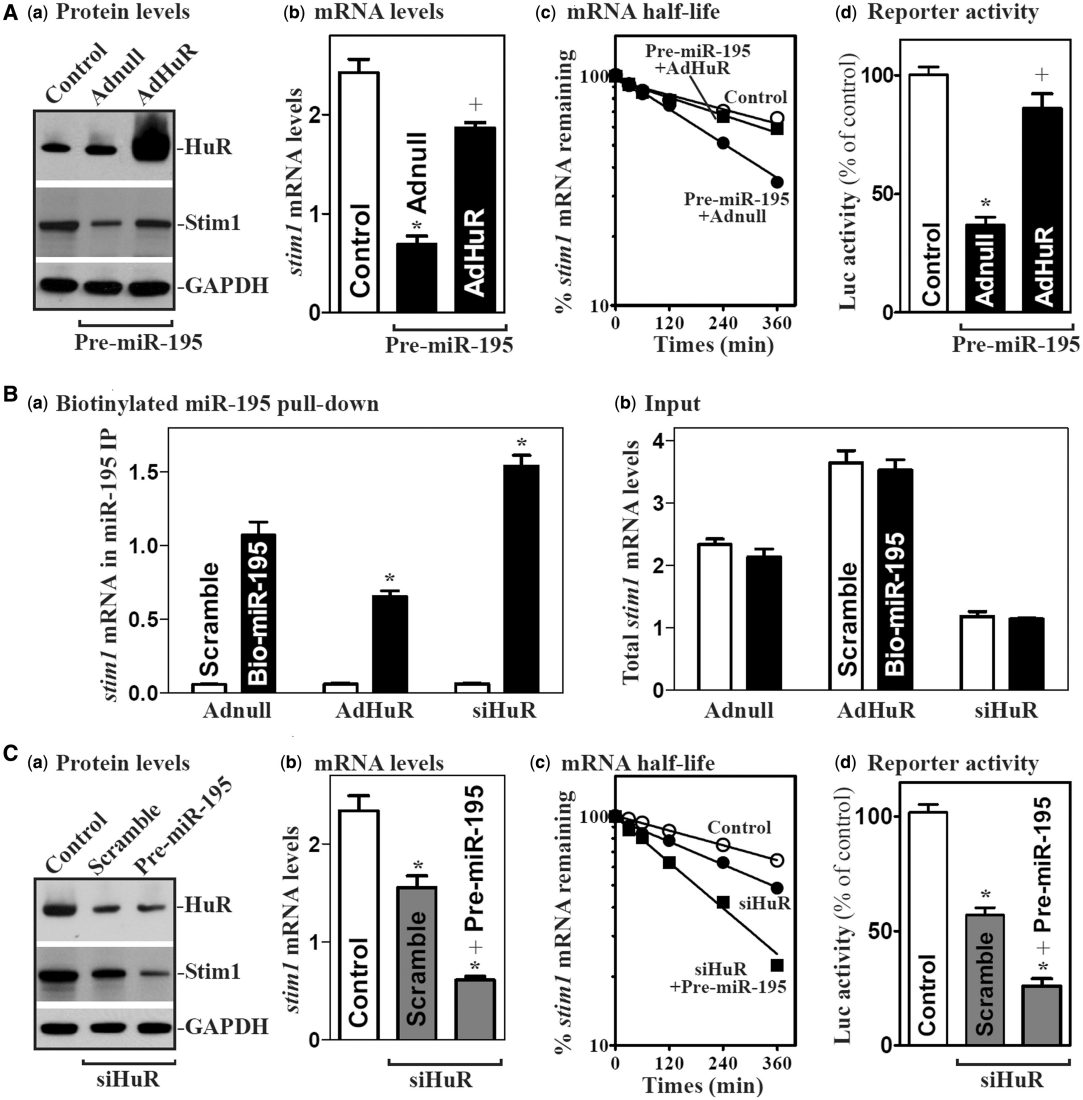
HuR and miR-195 regulate Stim1 expression antagonistically. (**A**) Effect of ectopic HuR overexpression on miR-195-mediated Stim1 repression: (**a**) HuR and Stim1 protein levels; (**b**) total *stim1* mRNA levels; (**c**) *stim1* mRNA stability; and (**d**) activities of *stim1* 3′UTR luciferase reporter. After cells were co-transfected with AdHuR and pre-miR-195 for 48 h, various measurements were performed. Values are the means ± SEM of data from three separate experiments. *P < 0.05 compared with controls. +*P* < 0.05 compared with cells transfected with pre-miR-195 and Adnull. (**B**) Binding of biotinylated miR-195 to the *stim1* mRNA as measured by Q-PCR analysis after cells were infected with AdHuR or transfected with siHuR for 48 h: (**a**) levels of mRNAs in the materials pulled down by biotin-miR-195; and (**b**) levels of total input mRNAs. *P < 0.05 compared with cells transfected with Adnull. (**C**) Effects of HuR silencing on miR-195-mediated Stim1 repression. Cells were co-transfected with siHuR and pre-miR-195 for 48 h; various measurements were performed as described earlier in the text. Values are the means ± SEM of data from three separate experiments. *^,+^*P* < 0.05 compared with controls and cells transfected with siHuR alone, respectively.

Given the fact that both miR-195 and HuR interact predominantly with the *stim1* 3′-UTR (Figures 3 and 4), we postulate that miR-195 and HuR might compete for association with *stim1* mRNA and thus regulate the stability of *stim1* mRNA antagonistically. To test this hypothesis, we first examined the effect of ectopic HuR overexpression on miR-195-induced repression of Stim1 expression. Ectopic HuR overexpression by infection with the AdHuR prevented the miR-195-mediated repression of Stim1 expression as indicated by an increase in the levels of Stim1 protein and mRNA (Figure 6Aa and b) in cells overexpressing miR-195. This effect resulted from an increase in stabilization of the *stim1* mRNA, as the decreased half-life of the *stim1* mRNA in pre-miR-195-transfected cells was completely prevented by HuR overexpression (Figure 6Ac). The stimulatory effect of HuR is mediated through its interaction with the *stim1* 3′-UTR as shown in results from luciferase reporter gene assays (Figure 6Ad). Increased HuR by AdHuR infection also decreased the levels of miR-195-*stim1* mRNA complex (Figure 6B), whereas HuR silencing by transfection with siHuR increased the amount of *stim1* mRNA associated with miR-195. Moreover, HuR silencing and miR-195 overexpression synergistically repressed Stim1 expression by enhancing degradation of the *stim1* mRNA (Figure 6C). In contrast, increased rate of *stim1* mRNA degradation in HuR-silenced cells was partially but significantly prevented by transfection with anti-miR-195 oligomer (Supplementary Figure S6). These results strongly suggest that HuR and miR-195 competitively bind to the *stim1* 3′-UTR and that HuR stabilizes *stim1* mRNA by displacing miR-195.

### Induced *stim1* mRNA recruitment to P-bodies by miR-195 is blocked by HuR

**Figure 7.**
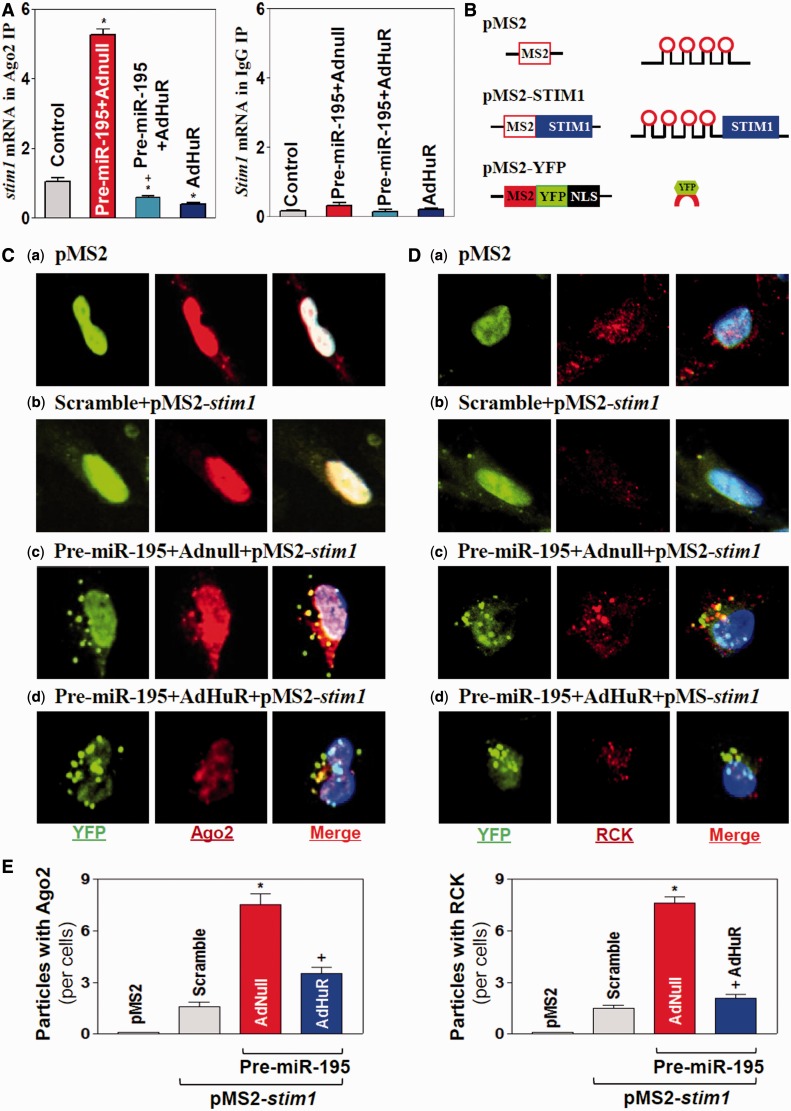
HuR prevents the miR-195-induced association of *stim1* mRNA with Ago2 and P-bodies. (**A**) *stim1* mRNA interaction with P-body components. After cell were transfected pre-miR-195 or infected by AdHuR alone or co-transfected with pre-miR-195 and AdHuR for 48 h, the association of *stim1* mRNA with Ago2 was measured by RNP-IP using anti-Ago2 antibody (*left*) or control IgG (*right*), which was followed by Q-PCR analysis. Values are the means ± SEM of data from three separate experiments. *^,+^*P* < 0.05 compared with control cells and cells transfected with pre-miR-195 alone, respectively. (**B**) Schematic of the plasmids used for the visualization of *stim1* mRNA. pMS2 and pMS2-stim1 expressed *MS2* and *MS2-stim1* RNAs, respectively, each containing 24 tandemMS2 hairpins; pMS2-YFP expressed a fusion fluorescent protein (MS2-YFP) capable of detecting MS2-containing RNA. (**C** and **D**) Images of *stim1* mRNA colocalization with P-bodies as indicated by its merging with Ago2 or RCK signals: (**a**) cells transfected with pMS2 alone; (**b**) cells transfected with scrambled siRNA and pMS2-Stim1; (**c**) cells transfected with pre-miR-195 and pMS2-Stim1; and (**d**) cells co-transfected with pre-miR-195 and AdHuR and with pMS2-Stim1. Confocal microscopy was used to visualize *MS2* and *MS2-stim1* mRNA using MS2-YFP (green fluorescence); red, Ago2 and RCK (P-body markers) signals; and yellow, colocalized red and green signals. Three experiments were performed and showed similar results. (**E**) Quantitative analysis of colocalization of *stim1* mRNA with P-body in controls (scramble), cells transfected with pre-miR-195 alone and cells co-transfected with pre-miR-195 and AdHuR. Values are the means ± SEM of data from 12 images. *^,+^*P* < 0.05 compared with scramble and pre-miR-195, respectively.

To define the mechanism by which miR-195 and HuR regulate *stim1* mRNA decay in opposite directions, we determined the role of association of the *stim1* mRNA with processing bodies (P-bodies). Increasing evidence indicates that non-translating mRNAs accumulate in P-bodies where these transcripts are believed to be sorted for degradation and/or translational repression (52–54). To determine whether miR-195 and HuR regulates *stim1* mRNA stability by altering *stim1* mRNA recruitment to P-bodies, we first examined changes in the *stim1* mRNA association with the P-body-resident protein Ago2 after ectopic miR-195 overexpression. As shown, miR-195 overexpression increased the association of *stim1* mRNA with P-bodies as indicated by an increase in the levels of *stim1* mRNA in the Ago2 IP (Figure 7A, *left*) compared with those observed in cells transfected with control scramble oligomer. Interestingly, the miR-195-induced association of *stim1* mRNA with Ago2 was completely prevented by HuR overexpression. Increasing HuR alone also decreased *stim1* mRNA presence in P-bodies, whereas HuR silencing increased the association of Ago2 with the *stim1* mRNA (Supplementary Figure S7A). As negative control, there were no changes in the levels of *stim1* mRNA in IgG IP materials between these groups. Furthermore, miR-195 did not repress Stim1 expression in the absence of Ago2 or RCK (another P-body resident protein), as silencing Ago2 or RCK prevented miR-195-induced repression of Stim1 expression (Supplementary Figure S7B and C). On the other hand, silencing Ago2 or RCK alone failed to repress Stim1 expression.

We further examined the subcytoplasmic localization of *stim1* mRNA by using plasmids that expressed MS2-tagged *stim1* mRNA. The reporter construct pMS2-Stim1, which expressed a chimeric RNA (*MS2-stim1*) comprising the *stim1* 3′-UTR and 24 tandem MS2 RNA hairpins (Figure 7B), was prepared as described previously (27,38). Co-transfection of pMS2-stim1 together with plasmid pMS2-YFP, which expressed the chimeric fluorescent protein MS2-YFP with a nuclear localization signal, allowed us to track the subcellular localization of the chimeric *MS2-stim1* RNA (as the complex MS2-YFP-*MS2-stim1*) as well as that of the control MS2 RNA (as the complex MS2-YFP-*MS2*) by confocal microscopy. To verify the functionality of our technique, the *MS2-stim1* reporter mRNA was shown to be efficiently immunoprecipitated with MS2-YFP using anti-YFP antibody (Supplementary Figure S8). Signals of the P-body markers Ago2 and RCK were also detected in the same cells. As shown, the control *MS2* RNA appeared to be exclusively nuclear in cells (Figure 7Ca and Da) owing to the presence of the nuclear localization signal in the chimeric protein MS2-YFP. Some *MS2-stim1* RNA was retained in the cytoplasm, colocalizing with some Ago2 (Figure 7Cb) and RCK signals (Figure 7Db) in cells transfected with the scrambled oligomer. However, miR-195 overexpression by pre-miR-195 transfection increased the co-localization of *MS2-stim1* RNA and Ago2 and RCK signals (colocalization results in yellow signals in the merged image in Figure 7Cc, Dc and E), suggesting that increasing the levels of miR-195 enhanced the association of *stim1* mRNA with P-bodies. Furthermore, the association of *MS2-stim1* RNA with P-bodies was lost when cells were co-transfected with the AdHuR and pre-miR-195 as indicated by a decrease in the colocalization of *MS2-stim1* RNA and Ago2 (Figure 7Cd and E, *left*) and RCK signals (Figure 7Dd and E, *right*). Together, these data support the notion that miR-195 enhances *stim1* mRNA decay at least partially by recruiting the *stim1* mRNA to P-bodies, whereas HuR prevents this recruitment, thus stabilizing the *stim1* mRNA.

### Control of *stim1* mRNA stability by miR-195 and HuR regulates epithelial repair

**Figure 8.**
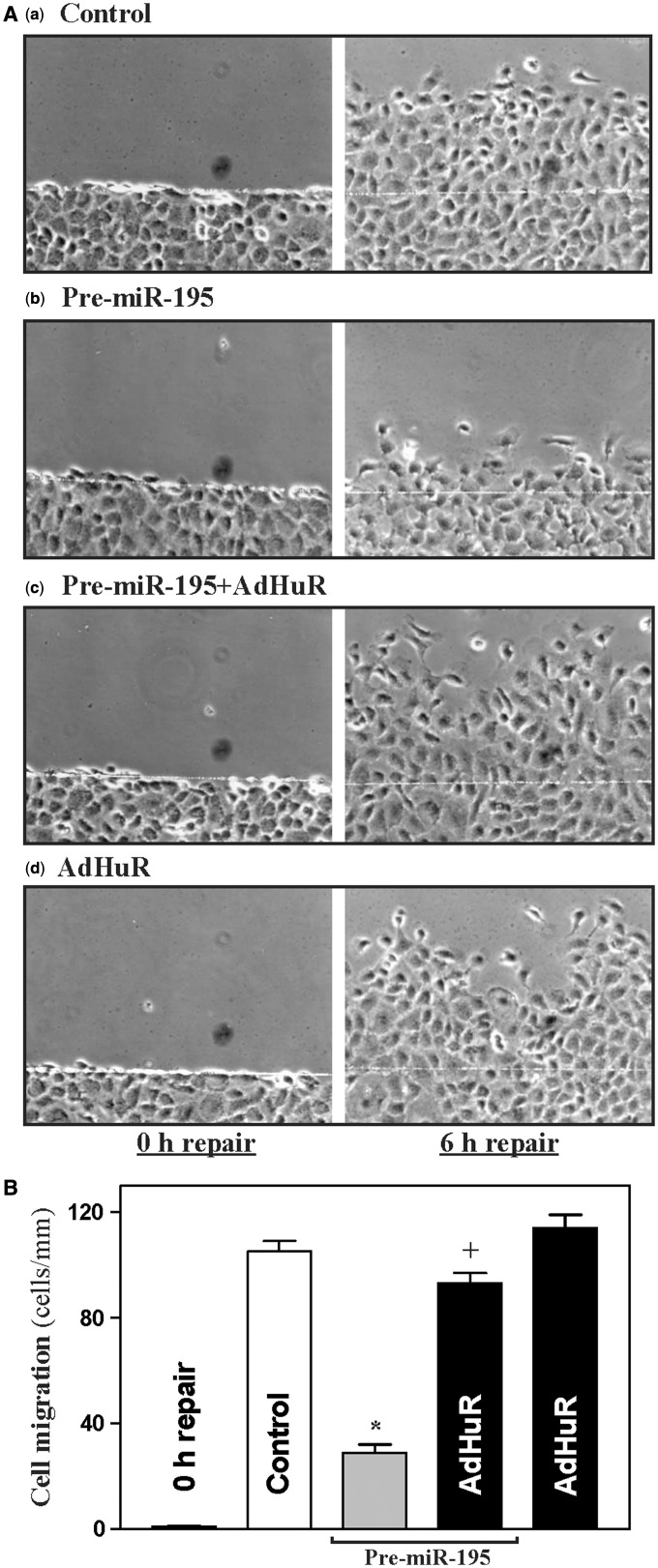
miR-195 inhibits intestinal epithelial repair after wounding. (**A**) Imaging pictures immediately after wounding (*left*, 0 h repair) and 6 h thereafter (*right*, 6 h repair): (**a**) controls; (**b**) cells transfected with pre-miR-195 alone; (**c**) cells co-transfected with pre-miR-195 and AdHuR; and (**d**) cells infected with AdHuR alone. After cells were transfected with pre-miR-195 or infected with AdHuR alone, or co-transfected with pre-miR-195 and AdHuR for 48 h, the monolayer was wounded by removing part of the monolayer as described in ‘Materials and Methods’ section. Plates were photographed immediately or 6 h after wounding. (**B**) Summarized data showing rates of cell migration 6 h after wounding in cells described in (**A**). Values are the means ± SEM of data from 6 dishes. *^,+^*P* < 0.05 compared with control and cells transfected with pre-miR-195 alone, respectively.

To investigate the biological significance of the miR-195/HuR-modulated Stim1 expression, we examined its involvement in the regulation of rapid epithelial repair after wounding. An *in vitro* repair model that mimics the early cell division-independent stage of epithelial restitution was used, as described in previous studies (5,50,55,56). As shown in Figure 8, early epithelial repair occurred quickly after wounding, as indicated by a significant increase in cell migration over the wounded area at 6 h. Cell division did not participate in this short process, as there were no changes in DNA synthesis within 6 h after wounding as measured by the technique of [^3^H]thymidine incorporation (data not shown). Furthermore, inhibition of cell proliferation by treatment with mitomycin C (2 µg/ml) also failed to alter cell migration when measured 6 h after wounding. Ectopic overexpression of miR-195 by pre-miR-195 transfection inhibited this early epithelial repair; the numbers of cells migrating over the wounded edge after wounding in pre-miR-195-transfected cells decreased significantly compared with control cells transfected with scramble. Importantly, increased HuR by AdHuR infection prevented miR-195-induced repression of epithelial repair, although it just marginally enhanced cell migration after wounding. These results show that control of stability of the *stim1* mRNA and subsequent Stim1 expression by miR-195 and HuR play a critical role in the regulation of rapid epithelial repair after wounding.

## DISCUSSION

Stim1 activates SOC channels such as TRPC1 and enhances Ca^2+^ influx after store depletion or in response to stressful environmental conditions including mucosal injury (11,12,14,15), but the exact mechanisms that control cellular Stim1 abundance remain unknown. In this study, we highlight the novel functions of miR-195 and RBP HuR in the regulation of *stim1* mRNA stability and provide insight into the control of Stim1 expression at the posttranscription level. As shown, *stim1* 3′-UTR is the target of both miR-195 and HuR, and they regulate *stim1* mRNA decay competitively and in opposite directions. miR-195 interacted with the *stim1* mRNA via its 3′-UTR and destabilized *stim1* transcript, whereas the stability of *stim1* mRNA increased with HuR association. Furthermore, miR-195 competed with HuR for association with the *stim1* 3′-UTR, and altering the interaction of HuR with *stim1* mRNA affected the levels of *stim1* mRNA associated with miR-195. These findings advance our understanding of the molecular mechanisms underlying the regulation of Stim1 gene expression and indicate that competitive binding of miR-195 and HuR to the *stim1* mRNA controls its stability and therefore modulates epithelial repair after wounding.

Results presented here show that miR-195 and HuR interacted with the *stim1* 3′-UTR, but not with its 5′-UTR or CR in IECs. Through the use of various ectopic reporters bearing partial transcripts spanning the *stim1* 3′-UTR with or without the miR-195 binding site, our results further show that the 2733–2753 site of the *stim1* 3′-UTR was the predominant site through which miR-195 destabilized *stim1* mRNA. These findings are consistent with other who results that miR-195 associated with the 3′-UTRs of *CDK4*, *CDK6*, *WEE1*, *MO25*, *ActRIIA* and *SIRT1* mRNAs (30–34,57) and thus represses their expression. In some instances, miRNAs were also shown to associate with the CRs of target mRNAs for their regulatory actions. In this regard, miR-503 represses *CUGBP1* mRNA translation by interacting with the *CUGBP1* CR rather than the 3′-UTR (26), and miR-519 inhibits HuR translation also through association with the *HuR* CR (23). Unlike miR-195 site, there were two HuR-hit motifs in the *stim1* 3′-UTR, which are located at the sequences spanning from 2435–2580 and 3780–3839, respectively (Supplementary Figure S5). These findings suggest that miR-195 and HuR interact with the *stim1* 3′-UTR through distinct and non-overlapping binding sites. We did not further characterize the specific *stim1* 3′-UTR nucleotides with which HuR interacts, as those experiments would require more specialized biochemical, crystallographic and molecular methods than those used here.

One important finding of this study is that miR-195 and HuR jointly regulate the *stim1* mRNA decay competitively and antagonistically. Increased HuR prevented the miR-195-induced destabilization of *stim1* mRNA by decreasing miR-195–*stim1* mRNA association, whereas HuR silencing enhanced the *stim1* mRNA decay induced by miR-195. Consistent with our current observations, several studies have also revealed that HuR antagonizes regulatory actions of given miRNAs. For example, HuR prevents miR-122-mediated repression of CAT-1 expression (58), antagonizes miR-548c-3p to regulate the expression of TOP2A (59), blocks miR-494 to regulate the expression of nucleolin (42) and co-operates with let-7 in repressing c-Myc expression (43). Although the exact mechanisms by which HuR competes with miRNAs for association with target transcripts remain unclear at present, it has been reported that miRNA binding sites are commonly present near HuR sites (60,61), suggesting that in some cases HuR and miRNAs could compete via their physical interaction with mRNAs. However, HuR and the coregulatory miRNAs can also bind at distances up to several hundreds or thousands of bases apart in some targets (43,58,59). In this study, the miR-195 site and two HuR sites are 153 and 1027 bases apart on the *stim1* 3′-UTR, respectively. It is clear that RNA structure and folding analyses will be needed to investigate systematically how binding of HuR in one area of the *stim1* 3′-UTR regulates interaction of miR-195 in a remote site and *vice versa*. Another possibility is that HuR may affect the processing pre-miR-195, but our preliminary studies showed that ectopic overexpression of HuR did not alter the levels of mature miR-195 in IECs.

Our results obtained in the present study also support the possibility that miR-195 and HuR jointly regulate the stability of *stim1* mRNA by altering the recruitment of *stim1* mRNA to P-bodies. Ectopic miR-195 overexpression increased *stim1* mRNA levels in P-bodies as measured by RNP IP using an antibody against the P-body resident proteins Ago2 and by measuring the colocalization of the *stim1* mRNA in P-bodies (Figure 7). In contrast, HuR overexpression decreased the levels of *stim1* mRNA associated with Ago2 and reduced the colocalization of *stim1* mRNA in P-bodies in cells overexpressing miR-195, indicating that HuR competes with miR-195 for the binding to *stim1* mRNA, thereby blocking induced *stim1* mRNA translocation to P-bodies by miR-195. P-bodies contain many proteins and enzymes that are associated with RNA-induced silencing complex and mRNA degradation, although the exact mechanism underlying mRNA decay within P-bodies is not fully known. These proteins and enzymes include all four human Ago proteins, GW182 (TNRC6A) together with its two human paralogues (TNRC6B and TNRC6C), two RNA helicases (RCK and MOV10), decapping enzymes (DCP1 and DCP2), mRNA deadenylation factors (such as the CCR4-CAF-1-Not complex), activators of decapping (Dhh1/RCK/p54, Pat1, Scd6/RAP55, Edc3 and the LSm1-7 complex) and exonucleases (such as XRN-1) (62,63). The mRNAs within P-bodies are thought to be subject to RNP remodeling to induce their subsequent turnover and to repress translational rates (52,62).

Finally, the miR-195- and HuR-regulated Stim1 expression is of biological significance because it plays an important role in the regulation of cell migration after wounding (Figure 8). Gut mucosal injury occurs in circumstances commonly encountered in daily life and in patients with critical illness, and the successful repair of damaged mucosa requires epithelial cell decisions that regulate signaling networks controlling expression of various genes involved in cell survival, apoptosis, migration and proliferation (64). Epithelial cells have to modify the patterns of expressed genes to repair damaged mucosa rapidly after injury. Early restitution is a critical repair modality, and it rapidly reseals superficial wounds by migrating visible remaining IECs from areas adjacent to, or just beneath, the injured surface to cover the wounded area, a process independent of cell proliferation (65,66). This rapid epithelial restitution is a complex process that is highly regulated by numerous extracellular and intracellular factors including Stim1/TRPC1-mediated Ca^2+^ signaling (5,14,15). Our current studies show that decreased levels of Stim1 by miR-195 overexpression repressed cell migration after wounding, and this effect was abolished by HuR overexpression. These findings provide additional new evidence that control of cellular Stim1 abundance by balancing the *stim1* mRNA associations with miR-195 and with HuR is crucial for normal epithelial restitution after wounding and thus for maintenance of the gut epithelial integrity.

## SUPPLEMENTARY DATA

Supplementary Data are available at NAR Online: Supplementary Tables 1 and 2 and Supplementary Figures 1–8.

## FUNDING

Merit Review Grants (to J-Y.W. and J.N.R.) from US Department of Veterans Affairs and from National Institutes of Health (NIH) [DK57819, DK61972, DK68491 to J-Y.W.]; National Institute on Aging-Intramural Research Program, NIH (to M.G.). Funding for open access charge: National Institute of Diabetes and Digestive and Kidney Diseases.

*Conflict of interest statement.* None declared.

## Supplementary Material

Supplementary Data
