# Supplementary material for: miR-195 competes with HuR to modulate stim1 mRNA stability and regulate cell migration
Source: Nucleic Acids Res. 2013 Jun 25;41(16):7905–19. doi: 10.1093/nar/gkt565 (PMC3763549; doi:10.1093/nar/gkt565)
Supplement: Supplementary Data [file supp_41_16_7905__index.html]

miR-195 competes with HuR to modulate stim1 mRNA stability and regulate cell migration — miR-195 competes with HuR to modulate stim1 mRNA stability and regulate cell migration — Supplementary Data 

# miR-195 competes with HuR to modulate *stim1* mRNA stability and regulate cell migration

## 

files

**Files in this Data Supplement:**

- Supplementary Data - pdf file
- Supplementary Data - pdf file
