# Supplementary material for: miR-195 competes with HuR to modulate stim1 mRNA stability and regulate cell migration
Source: Nucleic Acids Res. 2013 Jun 25;41(16):7905–19. doi: 10.1093/nar/gkt565 (PMC3763549; doi:10.1093/nar/gkt565)
Supplement: Supplementary Data [file supp_gkt565_nar-01253-x-2013-File010.pdf]

Table 1. Primers for STIM1 luciferase reporters

Name	sequence	Cutting site
pmirGLO-5UTR-F	CTCCCTCCCCCACCTCCGTG	N/A
pmirGLO-5UTR-B	CCATGACGGAAGGTCTCGGT	N/A
pmirGLO-CR-F	ATGGATGTGTGCGCTCGTCT	N/A
pmirGLO-CR-B	CTACTTCTTAAGAGGCTTCT	N/A
pmirGLO-3UTR-F	GCAGAATAGGGTGGTAGTAT	N/A
pmirGLO-3UTR-B	AAAATCTCCTACATGCTTTA	N/A
pmirGLO-F1-F	CCG**CTCGAG**GCAGAATAGGGTGGTAGTAT	*Xho* I
pmirGLO-F1-B	GC**TCTAGA**TCCCCACAACTGCTAGGAAC	*Xba* I
pmirGLO-F2-F	CCG**CTCGAG**CCTAGCAGTTGTGGGGAAGA	*Xho* I
pmirGLO-F2-B	GC**TCTAGA**AGGGGAGCAGAGGTAAGAGG	*Xba* I
pmirGLO-F3-F	CCG**CTCGAG**TGTGGGCTCCGAGGCAGTTG	*Xho* I
pmirGLO-F3-B	GC**TCTAGA**GGAGAAGTGGTCACACAGCA	*Xba* I
pmirGLO-F4-F	CTA**GCTAGC**TGCCTGCACAGCATCCTCCC	*Nhe* I
pmirGLO-F4-B	**ACGCGT**CGACAAAATCTCCTACATGCTTTA	*Sal* I
pMS2-3UTR-F	AGCAGTAAGCGGCCGCTCGAGGCAGAATAGGGTGGTAGTAT	N/A
pMS2-3UTR-B	CCCTCTAGATGCATGCTCGAGAAAATCTCCTACATGCTTTA	N/A

Table 2. Primers for STIM1 biotin pull down

NAME	SEQUENCE
STIM1-5UTR-bF	CCAAGCTTCTAATACGACTCACTATAGGGAGACTCCCTCCCCCACCTCCGTG
STIM1-5UTR-B	CCATGACGGAAGGTCTCGGT
STIM1-CR-bF	CCAAGCTTCTAATACGACTCACTATAGGGAGAATGGATGTGTGCGCTCGTCT
STIM1-CR-B	CTACTTCTTAAGAGGCTTCT
STIM1-3UTR-bF	CCAAGCTTCTAATACGACTCACTATAGGGAGAGCAGAATAGGGTGGTAGTAT
STIM1-3UTR-B	AAAATCTCCTACATGCTTTA
3UTR-F1-F	CCGCTCGAGGCAGAATAGGGTGGTAGTAT
3UTR-F1-B	GCTCTAGATCCCCACAACTGCTAGGAAC
3UTR-F2-bF	CCAAGCTTCTAATACGACTCACTATAGGGAGACCTAGCAGTTGTGGGGAAGA
3UTR-F2-F	CCGCTCGAGCCTAGCAGTTGTGGGGAAGA
3UTR-F2-B	GCTCTAGAAGGGGAGCAGAGGTAAGAGG
3UTR-F3-bF	CCAAGCTTCTAATACGACTCACTATAGGGAGATGTGGGCTCCGAGGCAGTTG
3UTR-F3-F	CCGCTCGAGTGTGGGCTCCGAGGCAGTTG
3UTR-F3-B	GCTCTAGAGGAGAAGTGGTCACACAGCA
3UTR-F4-bF	CCAAGCTTCTAATACGACTCACTATAGGGAGATGCCTGCACAGCATCCTCCC
3UTR-F4-F	CTAGCTAGCTGCCTGCACAGCATCCTCCC
3UTR-F4-B	ACGCGTCGACAAAATCTCCTACATGCTTTA
3UTR-F1-1-B	AGGGCAGGTATGTACAGTGC
3UTR-F1-2-F	CCAAGCTTCTAATACGACTCACTATAGGGAGAGGCCATGGAGGGGCTGGCCC
3UTR-F1-2-B	GGGAACAGCAACTAAGACAT
3UTR-F1-3-F	CCAAGCTTCTAATACGACTCACTATAGGGAGAGCTGCTGTCACATCCTCTCC
3UTR-F4-1-B	CTGGAAGAGGGGAGGCGTCA
3UTR-F4-2-F	CCAAGCTTCTAATACGACTCACTATAGGGAGATGAAGCTGCTGTGTGACCAC
3UTR-F4-2-B	CAGCCCCAGCTGCACCCAAC
3UTR-F4-3-F	CCAAGCTTCTAATACGACTCACTATAGGGAGATCTGTCATGCCTTTACCCTG
3UTR-F4-3delta-B	CCCATGTGAGGGTGTCCAGG
