# Supplementary material for: miR-195 competes with HuR to modulate stim1 mRNA stability and regulate cell migration
Source: Nucleic Acids Res. 2013 Jun 25;41(16):7905–19. doi: 10.1093/nar/gkt565 (PMC3763549; doi:10.1093/nar/gkt565)
Supplement: Supplementary Data [file supp_gkt565_nar-01253-x-2013-File011.pdf]

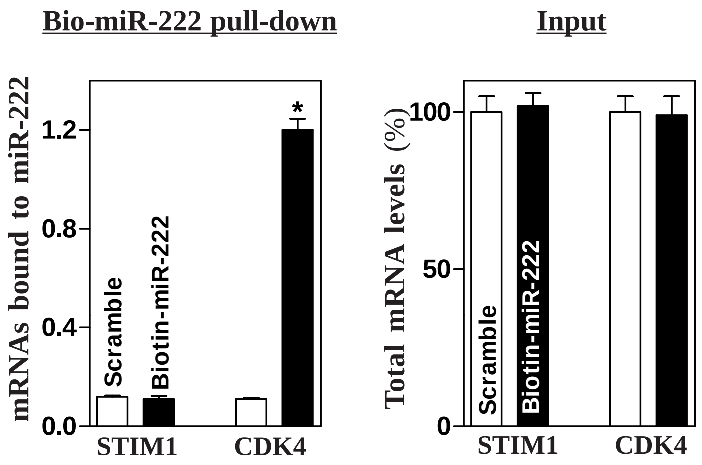

Supplemental Figure 1. Binding of biotinylated miR-222 to mRNAs encoding STIM1 and CDK4. *Right*, levels of mRNAs in the materials pulled down by biotin-miR-222 after 24 h transfection as measured by Q-PCR analysis. *Left*, levels of total input mRNAs. Values are the means ± SEM of data from three samples. * $p < 0.05$ compared with cells transfected with control scramble oligomer.

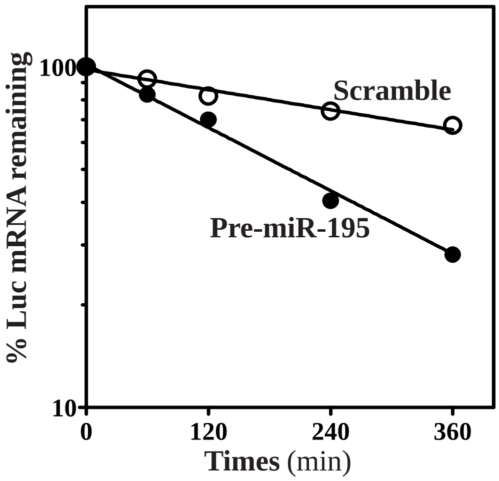

Supplemental Figure 2. The half-life of the luciferase mRNA expressed from the pmirGLO-STIM1-3'UTR luciferase reporter construct after transfection with the pre-miR-195. Total cellular RNA was isolated at indicated times after administration of actinomycin D, and the remaining luciferase and GAPDH mRNAs were measured by Q-PCR analysis. Values were means ± SE from triplicate experiments.

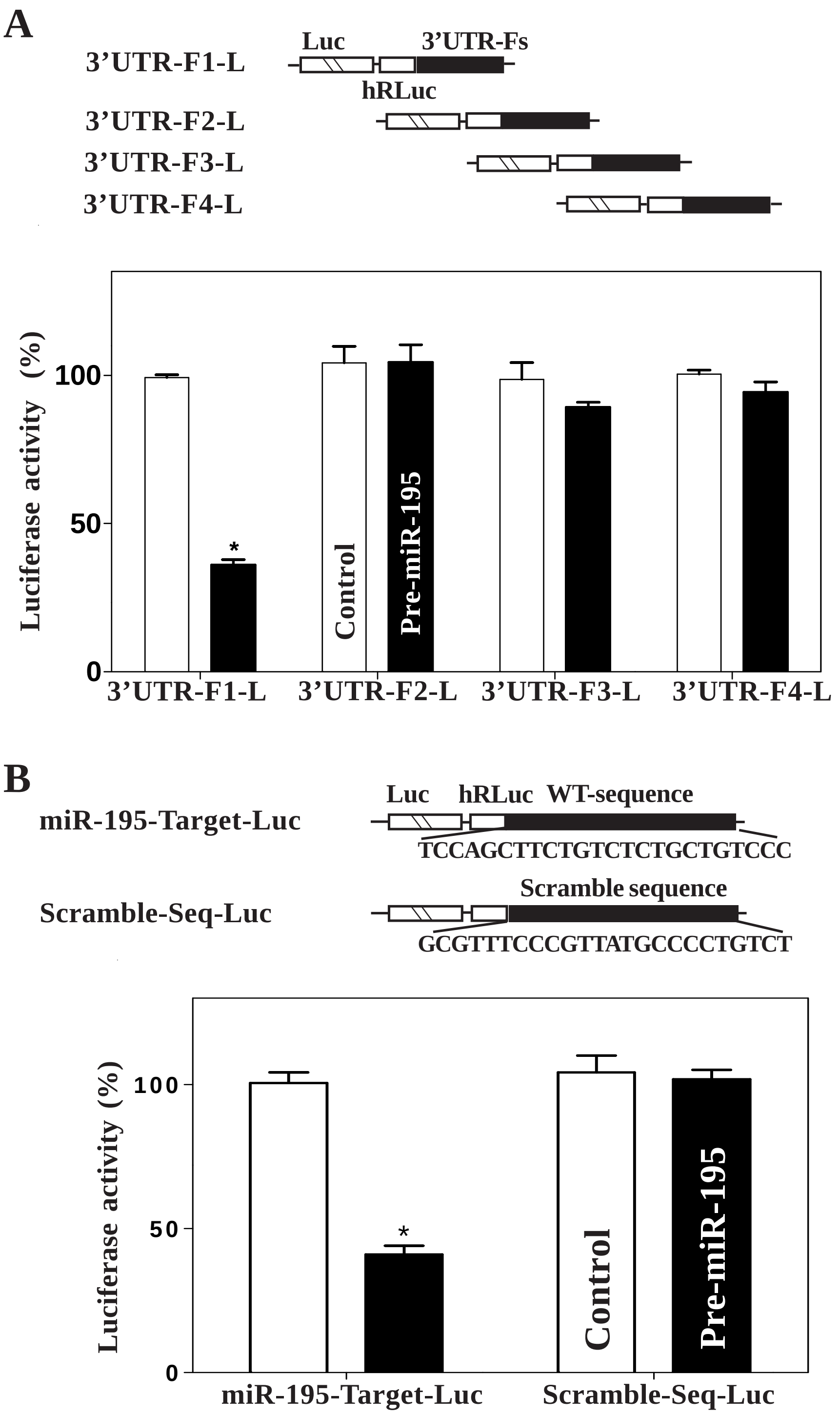

Supplemental Figure 3. Changes in activities of luciferase reporters containing fragments of Stim1 3’-UTR in cells overexpressing miR-195. (**A**) Levels of activities of Stim1 3’-UTR luciferase reporters. (**B**) Effect of mutation of miR-195-binding site (schematic) in Stim1 3'-UTR on luciferase reporter activities after ectopic miR-195 overexpression. Values are the means ± SEM of data from three samples. * $p < 0.05$ compared with cells transfected with control scrambled oligomer (control).

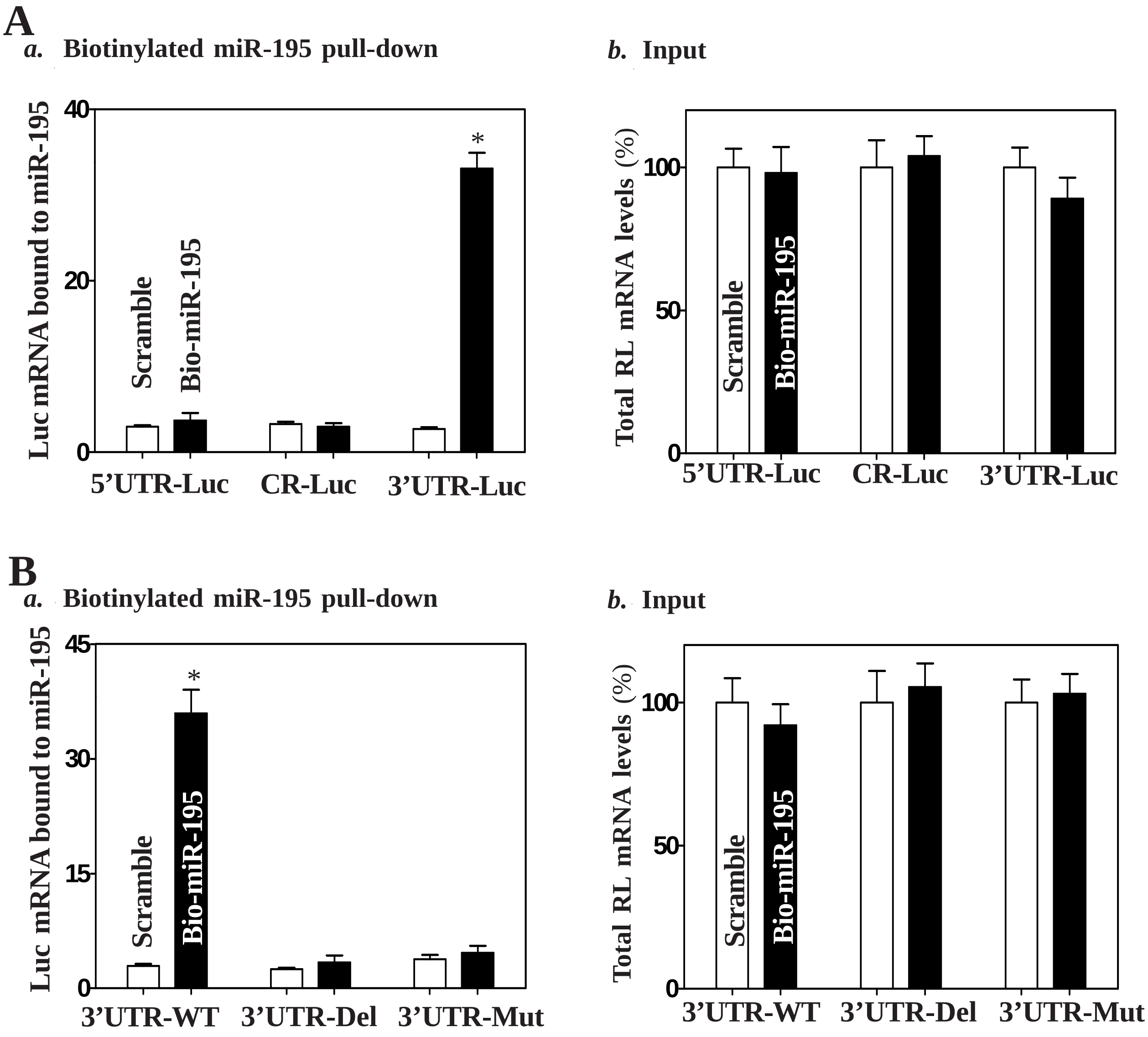

Supplement Figure 4. miR-195 specifically associates with the reporter mRNA containing the *Stim1* 3'-UTR. (**A**) Binding of biotinylated miR-195 to the reporter mRNA expressed from pmirGLO-STIM1-5’UTR, pmirGLO-STIM1-CR, or pmirGLO-STIM1-3’UTR constructs: (***a***) levels of the reporter mRNA in the materials pulled down by biotin-miR-195 and (***b***) levels of total input mRNAs. * $p < 0.05$ compared with cells transfected with control scramble oligomer. (**B**) Binding of biotinylated miR-195 to the reporter mRNA expressed the reporter constructs containing the wild type (WT) Stim1 3'-UTR or the Stim1 3'-UTR without the miR-195 binding site through deletion mutation and point mutation: (***a***) levels of the reporter mRNA in the materials pulled down by biotin-miR-195 and (***b***) levels of total input mRNAs. * $p < 0.01$ compared with cells transfected with control scramble oligomer.

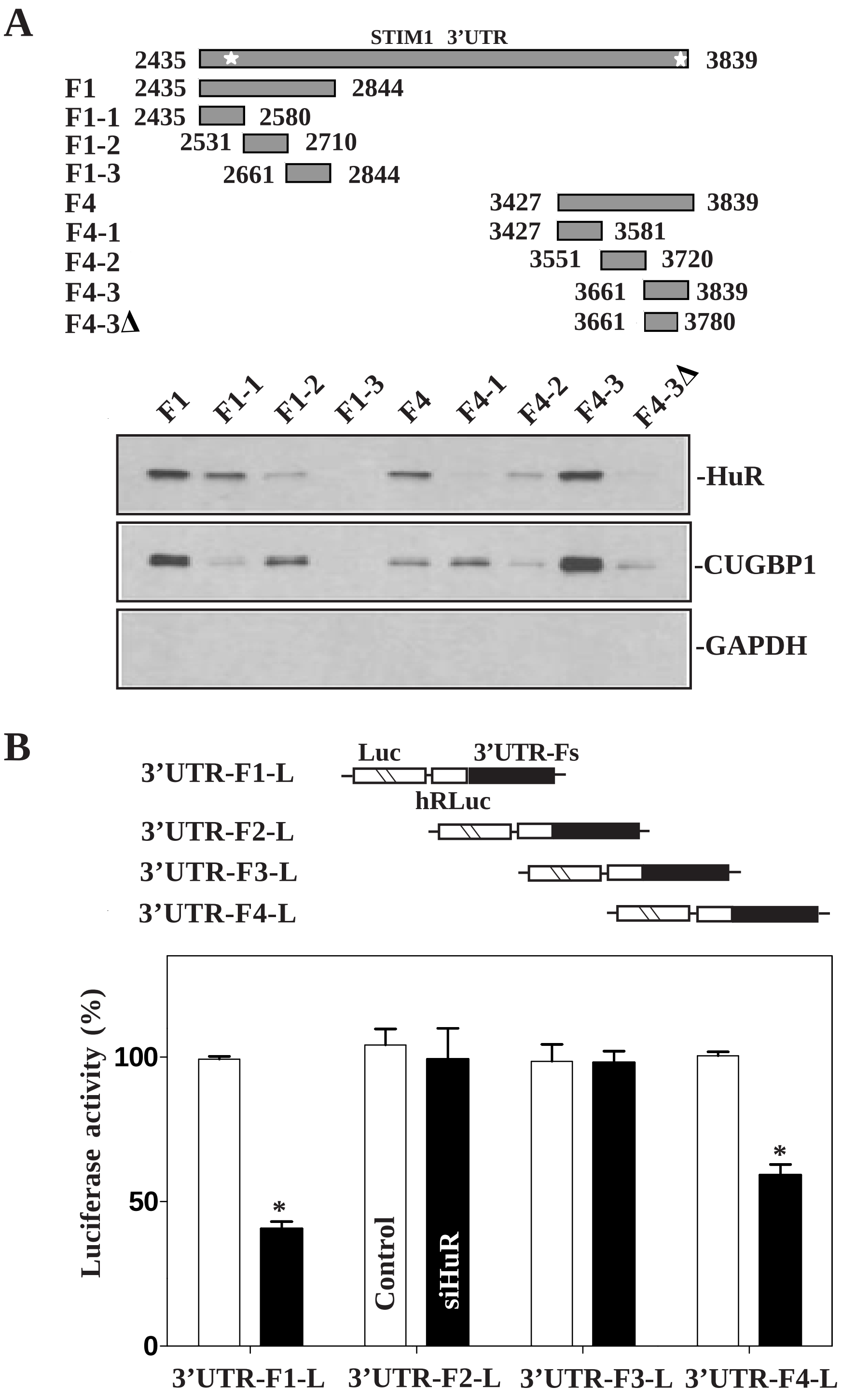

Supplemental Figure 5. HuR interacts with specific sequences of the Stim1 3'-UTR. (**A**) Representative HuR and CUGBP1 immunoblots after pull-down using biotinylated transcripts of different Stim1 3'-UTR fragments. Top panel, schematic representation of various biotinylated transcripts of the Stim 3'-UTR. (**B**) Changes in activities of luciferase reporter constructs containing various Stim 3'-UTR fragments in HuR-silenced cells. Top, schematic of plasmids of different chimeric firefly luciferase Stim1 3'UTR fragment reporters. Bottom, levels of luciferase reporter activities after cells were transfected with siHuR for 48 h. Values are the means ± SEM of data from three samples. * $p < 0.05$ compared with control siRNA (control).

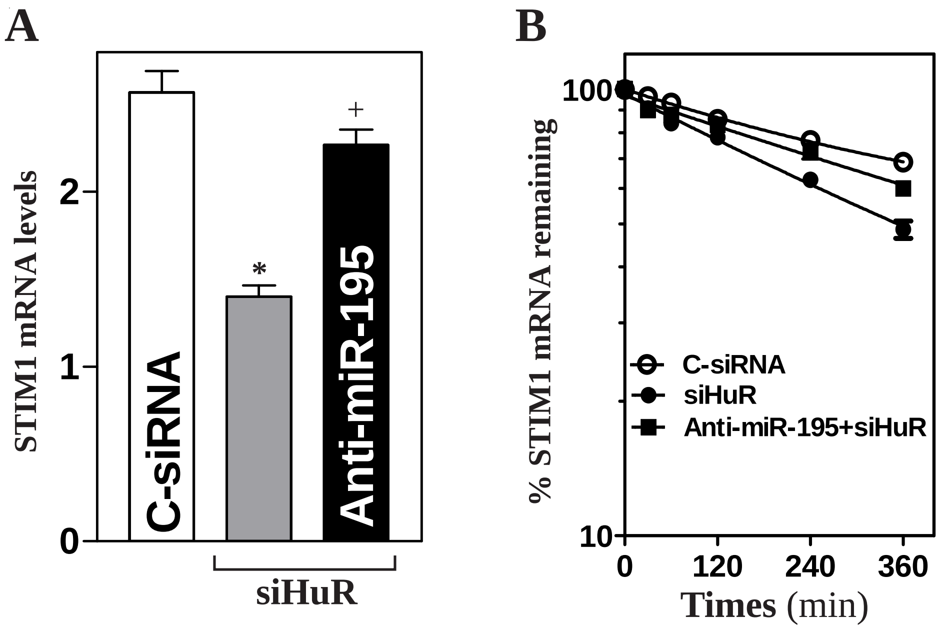

Supplement Figure 6. Effect of treatment with anti-miR-195 oligomer on the stability of the *Stim1* mRNA in HuR-silenced cells. (**A**) The levels of total *Stim1* mRNA in cells co-transfected with siHuR and anti-miR-195 for 48 h. Values are the means ± SEM of data from three separate experiments. $^{*,+}$ $P < 0.05$ compared with control siRNA (C-siRNA) and anti-miR-195, respectively. (**B**) The stability of the *Stim1* mRNA in cells described in (**A**). Values are the means ± SEM of data from three separate experiments.

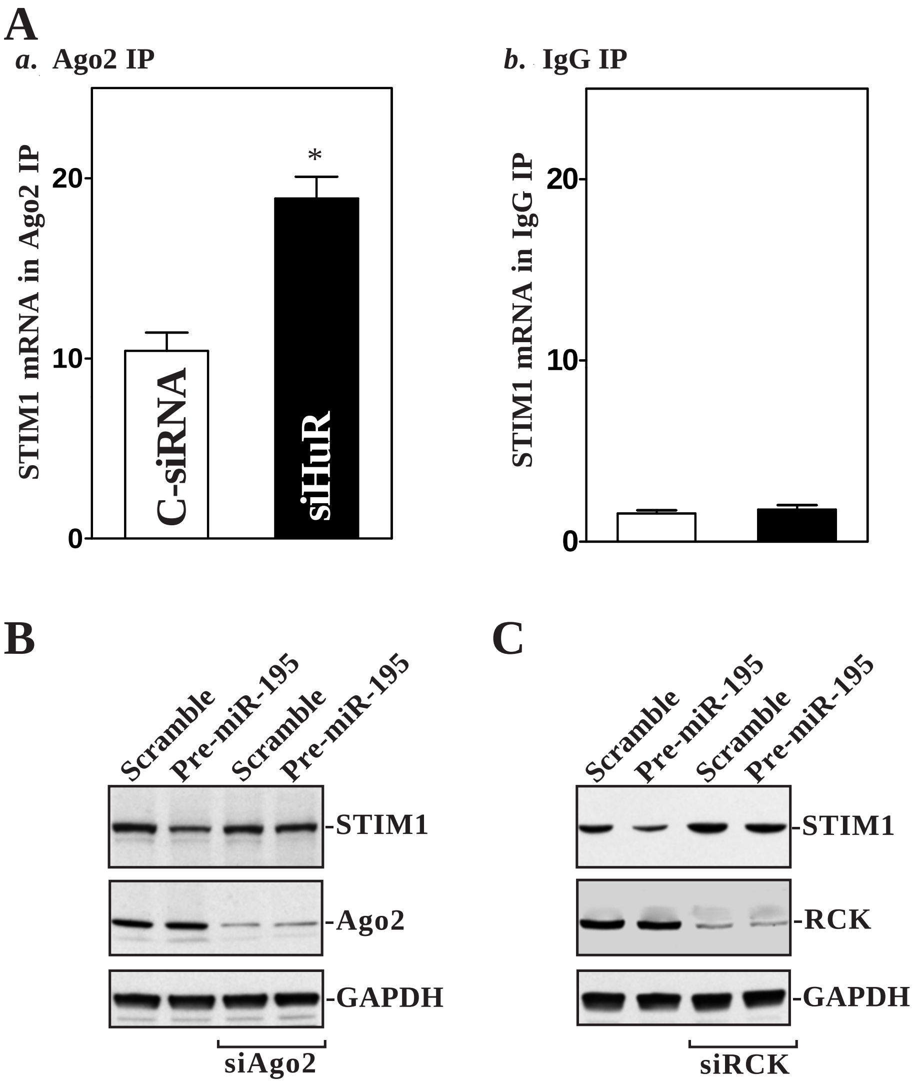

Supplement Figure 7. Effects of HuR, Ago2 and RCK on miR-195-induced repression of STIM1 expression. (**A**) Association of Ago2 with the Stim1 mRNA in HuR-silenced cells. After cell were transfected siHuR or C-siRNA for 48 h, the association of Ago2 with the Stim1 mRNA was measured by RNP-IP using anti-Ago2 antibody (***a***) or IgG (***b***), followed by Q-PCR analysis. Values are means ± SEM of data from three separate experiments. * $p < 0.05$ compared with C-siRNA. (**B**,**C**) Changes in STIM1 protein expression in cells overexpressing miR-195 in the presence or absence of Ago2 (B) or RCK (C). After cells were transfected with siAgo2 or siRCK for 24 h, they were transfected with the pre-miR-195. Levels of STIM1 and Ago2 or RCK proteins were measured 48 h after pre-miR-195 transfection.

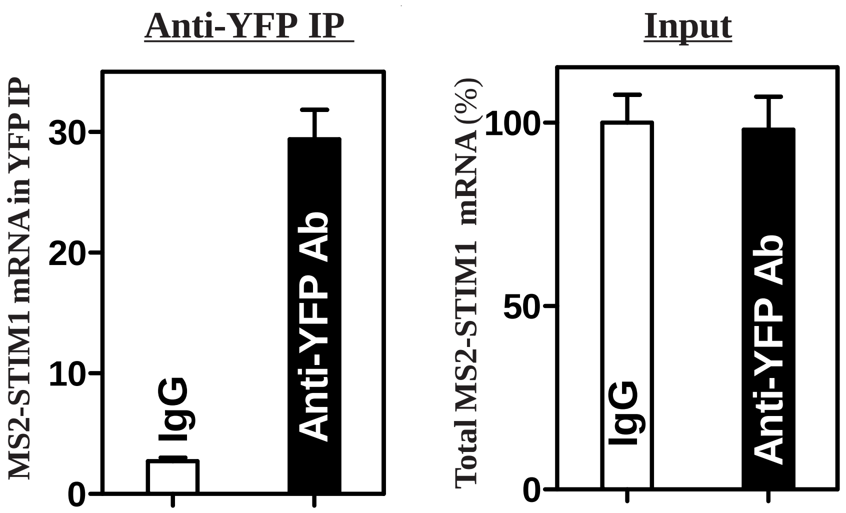

Supplement Figure 8. Association of the MS2-Stim1 reporter mRNA with the MS2-YFP. Forty-eight h after cells were co-transfected with pMS2-STIM1 reporter and pMS2-YFP vector, the association of the MS2-STIM1 reporter mRNA with MS2-YFP was measured by RNP/IP using anti-YFP antibody or IgG, followed by Q-PCR analysis. Values are the means ± SEM of data from three separate experiments.
